# Polycomb-Like 3 Promotes Polycomb Repressive Complex 2 Binding to CpG Islands and Embryonic Stem Cell Self-Renewal

**DOI:** 10.1371/journal.pgen.1002576

**Published:** 2012-03-15

**Authors:** Julie Hunkapiller, Yin Shen, Aaron Diaz, Gerard Cagney, David McCleary, Miguel Ramalho-Santos, Nevan Krogan, Bing Ren, Jun S. Song, Jeremy F. Reiter

**Affiliations:** 1 Department of Biochemistry and Biophysics, Cardiovascular Research Institute, University of California San Francisco, San Francisco, California, United States of America; 2 Ludwig Institute for Cancer Research, School of Medicine, University of California San Diego, San Diego, California, United States of America; 3 Institute for Human Genetics, University of California San Francisco, San Francisco, California, United States of America; 4 School of Biomolecular and Biomedical Science, University College Dublin, Dublin, Ireland; 5 Department of Obstetrics, Gynecology, and Reproductive Sciences, University of California San Francisco, San Francisco, California, United States of America; 6 Department of Cellular and Molecular Pharmacology, University of California San Francisco, San Francisco, California, United States of America; 7 Department of Biostatistics and Epidemiology, Department of Bioengineering and Therapeutic Sciences, University of California San Francisco, San Francisco, California, United States of America; Centre National de la Recherche Scientifique, France

## Abstract

Polycomb repressive complex 2 (PRC2) trimethylates lysine 27 of histone H3 (H3K27me3) to regulate gene expression during diverse biological transitions in development, embryonic stem cell (ESC) differentiation, and cancer. Here, we show that Polycomb-like 3 (Pcl3) is a component of PRC2 that promotes ESC self-renewal. Using mass spectrometry, we identified Pcl3 as a Suz12 binding partner and confirmed Pcl3 interactions with core PRC2 components by co-immunoprecipitation. Knockdown of *Pcl3* in ESCs increases spontaneous differentiation, yet does not affect early differentiation decisions as assessed in teratomas and embryoid bodies, indicating that Pcl3 has a specific role in regulating ESC self-renewal. Consistent with Pcl3 promoting PRC2 function, decreasing Pcl3 levels reduces H3K27me3 levels while overexpressing Pcl3 increases H3K27me3 levels. Furthermore, chromatin immunoprecipitation and sequencing (ChIP-seq) reveal that Pcl3 co-localizes with PRC2 core component, Suz12, and depletion of Pcl3 decreases Suz12 binding at over 60% of PRC2 targets. Mutation of conserved residues within the Pcl3 Tudor domain, a domain implicated in recognizing methylated histones, compromises H3K27me3 formation, suggesting that the Tudor domain of Pcl3 is essential for function. We also show that Pcl3 and its paralog, Pcl2, exist in different PRC2 complexes but bind many of the same PRC2 targets, particularly CpG islands regulated by Pcl3. Thus, Pcl3 is a component of PRC2 critical for ESC self-renewal, histone methylation, and recruitment of PRC2 to a subset of its genomic sites.

## Author Summary

Embryonic development requires coordinated changes in gene expression for the differentiation of specific cell types. Regulated changes in gene expression are also important for maintaining tissue homeostasis and preventing cancer. Histone modifications contribute to the control of gene expression by affecting chromatin structure and the recruitment of regulatory proteins. Polycomb repressive complex 2 (PRC2) catalyzes the methylation of a lysine residue on histone H3, an early step in gene repression. By investigating how PRC2 is recruited to genes, we have found that Polycomb-like 3 (Pcl3), a protein upregulated in diverse cancers, is a component of PRC2 that promotes its binding and function at target genes. Consistent with roles for PRC2 in regulating stem cell behaviors, Pcl3 is important for embryonic stem cell self-renewal. Thus, Pcl3 is a critical regulator of gene repression and stem cell self-renewal that acts by controlling PRC2 binding to target genes.

## Introduction

The developmental plasticity of early embryos and embryonic stem cells (ESCs) requires the repression of cell-type specific genes. Two multiprotein complexes that participate in gene repression are Polycomb repressive complex 1 (PRC1) and Polycomb repressive complex 2 (PRC2) [1], [2]. Core components of PRC2 include Suz12, Eed, and Ezh2, a methyltransferase that participates in di- and tri-methylation of lysine 27 on histone H3 (H3K27me2/3) [2]–[7]. Trimethylation of H3K27 can modulate the function of PRC1, which mono-ubiquitinates histone H2A on lysine 119 (H2AK119Ub) [3], [4]. Both H3K27me3 and H2AK119Ub are early histone modifications involved in gene repression [7].

Whereas H3K27me3 is associated with repressed genes, H3K4me3 marks active genes. ESCs and a number of adult stem cells, however, contain a unique chromatin signature, termed bivalency, that is comprised of both H3K27me3 and H3K4me3 marks [8]–[15]. Many bivalent domains are at CpG islands, domains of DNA with elevated GC content that display low levels of DNA methylation. CpG islands are commonly found at vertebrate promoters and are associated with 70% of annotated genes including most housekeeping genes and many developmentally regulated genes [16]–[18]. CpG-rich domains commonly display H3K4me3, but GC-rich sequences also promote H3K27me3, creating opposing marks within the same domain [19], [20]. By occupying CpG islands and marking them as bivalent domains in ESCs, PRC2 may keep the associated genes repressed but poised for rapid activation upon differentiation [11]. How PRC2 is recruited to CpG islands is not known.

Disrupting core components of PRC2 causes a global reduction of H3K27me3 and misexpression of repressed genes, particularly bivalent genes [11], [21]–[24]. This dysregulation of gene expression perturbs ESC maintenance and differentiation, and results in embryonic lethality in mice [23], [25]–[30]. Furthermore, expression of PRC1 and PRC2 components is misregulated in diverse cancers, suggesting that PRC2-dependent gene regulation protects against neoplasia [31]–[34].

Beyond the core components of PRC2, accessory proteins such as Aebp2, Rbbp4/7, and Jarid2, influence PRC2 function [3], [35]–[40]. Recently, Polycomb-like (Pcl) proteins, named for the similarity of the Drosophila *Pcl* mutant phenotype to that of the *Polycomb* mutant, have been found to modulate PRC2 activity [41]–[48]. Drosophila Pcl has three homologs in mammals: Pcl1 (also called PHD finger protein 1), Pcl2 (also called Metal response element binding transcription factor 2), and Pcl3 (also called PHD finger protein 19) [49]. *Pcl1* is expressed minimally in ESCs, but promotes PRC2 function in adult tissues and male germ cells [41], [50]–[52]. Pcl2 regulates PRC2 differentially depending on cell context and target. In mouse embryonic fibroblasts (MEFS), Pcl2 inhibits PRC2 activity, whereas in ESCs, Pcl2 hinders H3K27me3 formation globally but promotes PRC2 activity at a subset of genes [41], [47], [48]. Human Pcl3 exists as two isoforms, which can bind Ezh2 and Eed [53]. Mammalian Pcl3 is expressed in ESCs, but it has been unclear how it contributes to PRC2 function and ESC biology.

Here, we show that mouse Pcl3 interacts with the core components of PRC2 and promotes complex function. By depleting Pcl3 in ESCs, we demonstrate that Pcl3 contributes to ESC self-renewal, but not differentiation, of the three germ layers. Using ChIP-seq of *Pcl3* shRNA-treated cells, we show that *Pcl3* knockdown causes decreased H3K27me3 and Suz12 binding to the genome, indicating that Pcl3 regulates PRC2 binding at diverse target genes. Furthermore, Pcl3 localizes with Suz12 at a subset of PRC2 targets, including genes and microRNAs associated with differentiation and development. We also show that several Pcl3 Tudor domain residues are necessary for H3K27me3. Finally, we identify two GC-rich binding motifs that are enriched at Pcl3-dependent PRC2 targets, indicating that Pcl3 promotes PRC2 binding at CpG islands. Taken together, these results reveal that Pcl3 is an important regulator of PRC2 at a subset of target genes.

## Results

### Pcl3 is a component of PRC2

**Figure 1:**
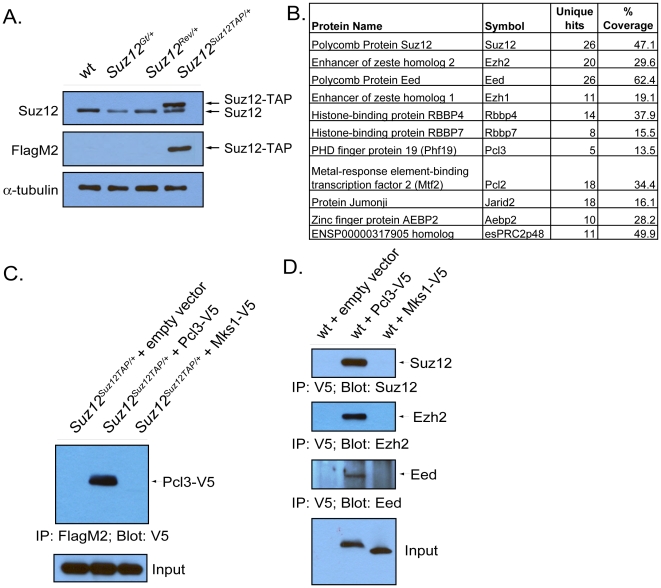
Pcl3 is a component of PRC2. (A) Protein levels of Suz12 and Suz12-TAP were measured in wild type, *Suz12^Gt/+^*, *Suz12^Rev/+^*, and *Suz12^Suz12TAP/+^* cell lines by immunoblot. (B) Proteins detected by mass spectrometry that specifically co-purified with Suz12-TAP, their symbol, unique hits, and percent coverage. (C) Pcl3-V5 binds to Suz12-TAP. *Suz12^Suz12TAP/+^* ESCs were transfected with empty vector, Pcl3-V5, or Mks1-V5 (control), and lysates were immunoprecipitated with FlagM2 and probed with anti-V5. (D) Pcl3-V5 binds Suz12, Ezh2, and Eed. Lysates from ESCs transfected with empty vector, Pcl3-V5, and Mks1-V5 (control) vectors were subjected to immunoprecipitation with anti-V5. Samples were then probed with anti-Suz12, anti-Ezh3, and anti-Eed. All westerns and co-immunoprecipitations were performed three times.

To identify PRC2 binding partners that could contribute to its function, we used the recently developed Floxin system to create a tandem affinity purification (TAP) tagged allele of *Suz12* (Figure S1A) [54]. In brief, we reverted a *Suz12* gene trap (*Suz12^Gt/+^*) allele generated in a mouse ESC line to produce an allele that re-expresses *Suz12* but that contains a loxP targeting site (*Suz12^Rev/+^*). Via a modified Floxin shuttle vector, we inserted an exon encoding amino acids 277–741 of Suz12 fused to a carboxy-terminal 6×His-3×Flag TAP tag. The resultant allele (*Suz12^Suz12TAP/+^*) expressed the full-length TAP-tagged Suz12 from the endogenous locus (Figure 1A and Figure S1B). We measured protein and mRNA levels of Suz12 in all ESC lines by immunoblot and quantitative reverse transcription PCR (qRT-PCR) (Figure 1A and Figure S1C). As expected, *Suz12^Gt/+^* cells displayed reduced Suz12 expression, whereas *Suz12^Rev/+^* cells displayed levels restored to wild type amounts (Figure 1A and Figure S1C). *Suz12^Suz12TAP/+^* cells displayed moderately increased mRNA and protein levels of Suz12 compared to wild type (Figure 1A and Figure S1C).

To reveal novel binding partners of Suz12, we tandem affinity purified Suz12-TAP from *Suz12^Suz12TAP/+^* ESCs and identified co-purified proteins by mass spectrometry (Figure 1B) [41]. The PRC2 core components Eed, Ezh1, and Ezh2 were highly represented among Suz12 co-purified proteins, as were other known PRC2 interactors, including Rbbp4, Rbbp7, Aebp2, Jarid2, Pcl2, and esPRC2p48 [3], [35]–[41], [47], [48]. In addition, we identified Pcl3 as co-purifying with Suz12. Human Pcl3 contains a long and short isoform, both of which are associated with PRC2 in HEK 293 cells by gel filtration chromatography [53]. To verify binding of mouse Pcl3 with PRC2, we confirmed that V5-tagged Pcl3 co-immunoprecipitated with Suz12-TAP in ESCs (Figure 1C and Figure S1D). To determine whether Pcl3 interacts with all PRC2 core members, we immunoprecipitated Pcl3-V5 and probed for Suz12, Ezh2, and Eed. All core components of PRC2 were found to bind Pcl3 (Figure 1D). Thus, mass spectrometric and co-immunoprecipitation analyses indicated that Pcl3 interacts with PRC2.

### Pcl3 promotes ESC self-renewal

Inhibiting PRC2 activity can affect both ESC self-renewal and differentiation by deregulating cell-type specific genes [22], [24]–[27], [29], [30]. *Suz12^−/−^* ESCs cannot form neural lineages, whereas *Eed^−/−^* ESCs show an increased propensity to differentiate [23], [24], [29], [55]. To determine if Pcl3 regulates ESC maintenance or differentiation, we tested whether *Pcl3* knockdown altered the ability of ESCs to self-renew or generate cell types derived from all three germ layers.

**Figure 2:**
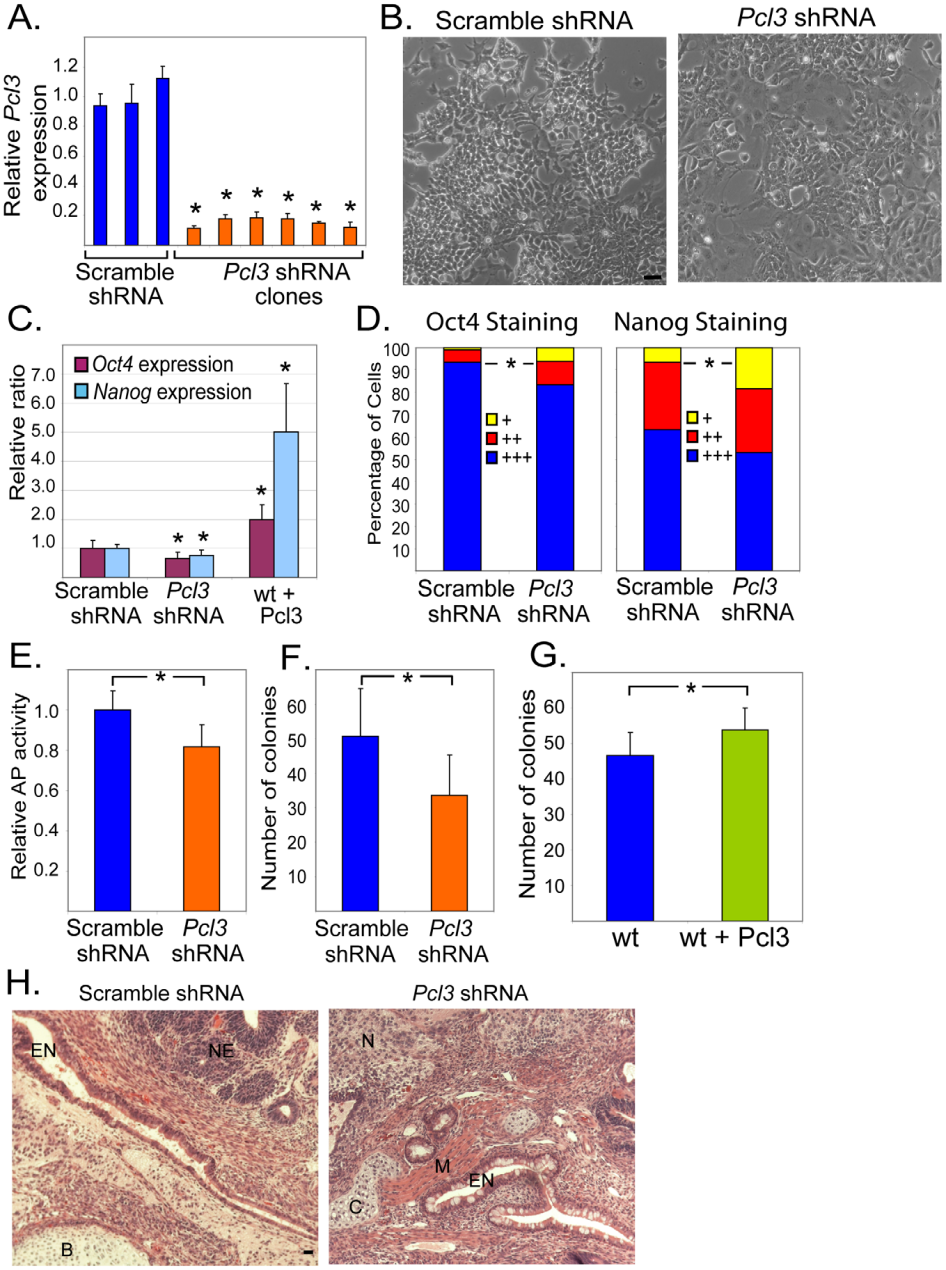
Pcl3 promotes ESC self-renewal. (A) *Pcl3* expression levels measured by qRT-PCR in ESC clones transduced with scramble or multiple *Pcl3* shRNAs. Graph represents average expression from 3–6 different clones. (B) A portion of cells transduced with *Pcl3* shRNA, but not scramble shRNA, are larger, flatter, and less dense, signifying a decrease in ESC cell morphology. Scale bar 25 µm. These pictures are representative of 2–3 different clones of scramble and *Pcl3* shRNA cells taken at three different time points. (C) Expression levels of *Oct4* and *Nanog* in scramble, *Pcl3* shRNA, and *Pcl3* overexpressing cells. (D) Quantification of Oct4 and Nanog staining in scramble and *Pcl3* shRNA treated ESCs. +++indicates bright staining, ++indicates less bright staining, and+indicates little or no staining as assessed by eye. Graphs are representative of two clones and between 5–10 fields of view at 10× magnification. (E) Alkaline phosphatase activity in scramble and *Pcl3* shRNA cells. Graph represents average activity from 3–6 different clones in three experiments assayed in duplicate. (F) Quantification of the number of colonies formed per well from scramble and *Pcl3* shRNA cells plated at 100 cells/well in a 6-well plate. Experiment was performed four times in duplicate with two clones each of scramble and *Pcl3* shRNA ESCs. (G) Quantification of colonies formed by plating 100 cells/well of wild type and Pcl3 overexpressing cells in a 6-well plate. LIF was reduced to 5% and was performed four times in duplicate. (H) Images of teratomas derived from scramble or *Pcl3* shRNA ESCs containing all three germ layers stained with hematoxylin and eosin. Abbreviations: EN-endoderm, NE-neuroectoderm, B-bone, C-cartilage, M-muscle, N-neural tissue. Scale bar 25 µm. Error bars indicate standard deviation. Expression analysis experiments represent 3–4 experiments assayed in quadruplet. For all experiments, asterisk denotes statistical significance of *p*<0.05. Staining was performed 2–3 times in two or more clones.

We depleted Pcl3 using multiple *Pcl3* shRNA targeting vectors (Figure 2A and Figure S2A). Upon culturing multiple clones of *Pcl3* knockdown ESCs, we observed an increased percentage of cells that were larger, less dense and displayed morphologies consistent with differentiation (Figure 2B). These differentiated morphologies suggested that Pcl3 may play a role in ESC maintenance or self-renewal.

To assess whether Pcl3 contributes to ESC self-renewal, we examined ESC markers including Oct4, Nanog, and alkaline phosphatase. Consistent with the morphological changes, Oct4 and Nanog expression and protein levels were decreased in *Pcl3* knockdown cells compared to control ESCs (Figure 2C–2D and Figure S2B). We also assessed scramble and *Pcl3* shRNA ESCs for alkaline phosphatase activity. Alkaline phosphatase staining was slightly reduced in *Pcl3* shRNA-treated ESCs (Figure S2C). To quantitate this observation, we used a more sensitive colorimetric assay, which confirmed that *Pcl3* knockdown reduces ESC alkaline phosphatase activity (Figure 2E). To confirm that these phenotypes were specifically due to *Pcl3* knockdown, we overexpressed a TAP-tagged form of Pcl3 in wild type cells (Figure S2D–S2E). Overexpression of *Pcl3* in ESCs resulted in increased levels of *Oct4* and *Nanog*, further indicating that Pcl3 levels correlate with ESC gene expression (Figure 2C).

To determine whether decreased expression of *Oct4* and *Nanog* affects self-renewal, we assessed *Pcl3* knockdown ESCs for their ability to generate colonies and found that *Pcl3* shRNA ESCs formed significantly fewer colonies than control cells (Figure 2F and Figure S2F). These data suggest that Pcl3 promotes ESC self-renewal. To substantiate this finding, we assayed the ability of wild type and *Pcl3*-overexpressing cells to self-renew and form colonies. We challenged wild type and *Pcl3*-overexpressing cells by growing them in media containing reduced LIF. *Pcl3*-overexpressing ESCs were able to form colonies modestly but significantly better than wild type cells, further indicating that Pcl3 enhances ESC self-renewal (Figure 2G).

In addition to its roles in self-renewal, PRC2 is critical for ESC differentiation and embryonic development [22], [24], [29], [30]. Besides ESCs, *Pcl3* is expressed in a number of differentiated tissues from all three germ layers including bone, spleen, and prostate [41]. *Pcl3* is not well expressed in the adult nervous system, but it expressed in the head during development [41]. To ascertain whether Pcl3 is essential for ESC differentiation, we performed teratoma and *in vitro* differentiation assays and assessed the presence of all three germ layers. Teratomas from scramble and *Pcl3* shRNA-expressing cells were weighed and histologically examined. While *Pcl3* knockdown was maintained, we did not observe differences in the size of the teratomas nor in the formation of endodermal, ectodermal, or mesodermal derivatives (data not shown and Figure 2H and Figure S2G). Moreover, immunofluorescent staining revealed the presence of neural tissue (NeuN and Tuj1), muscle (Actin), and skin basal cells (K14) in both *Pcl3* expressing and *Pcl3* knockdown teratomas (Figure S2H).

*In vitro* differentiation of scramble and *Pcl3* knockdown ESCs to embryoid bodies resulted in a similar finding. *Pcl3* shRNA-treated EBs maintained *Pcl3* knockdown and expressed markers of neuroectoderm (*Nestin*), mesoderm (*T-brachyury*), and endoderm (*Hnf4*) at least as well as control EBs, indicating that depletion of *Pcl3* does not abrogate the ability of ESCs to differentiate to cell types of all three germ layers (Figure S2I). Thus, Pcl3 enhances ESC self-renewal, but is not critical for ESC differentiation to the three germ layers.

### Pcl3 promotes trimethylation of H3K27

**Figure 3:**
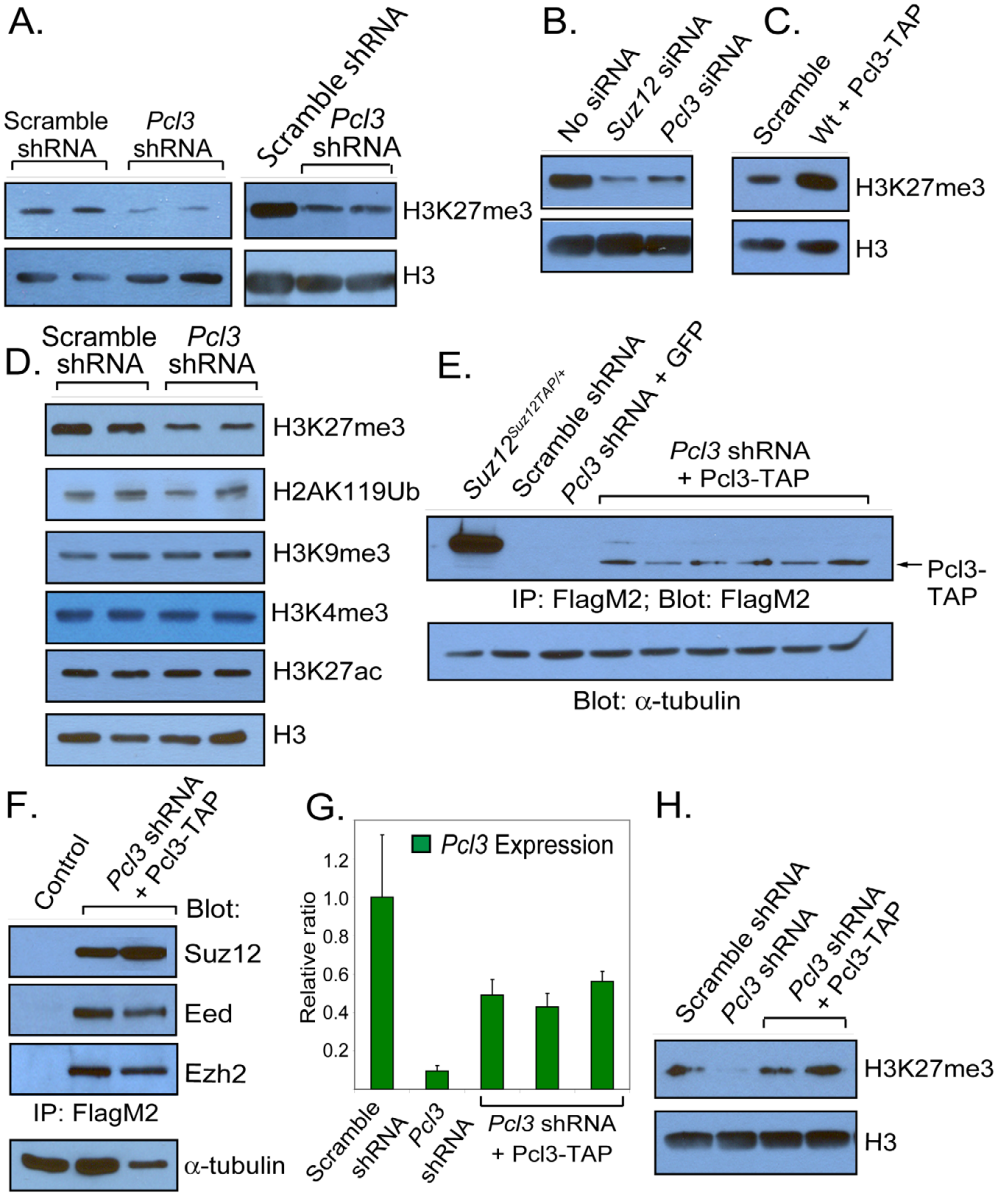
Pcl3 promotes PRC2 function. (A) Immunoblot showing levels of H3K27me3 in multiple clones of scramble and *Pcl3* shRNA ESCs and EBs. (B) H3K27me3 levels in *Suz12* and *Pcl3* siRNA treated cells. (C) Increased levels of H3K27me3 as measured by immunoblot in cells overexpressing Pcl3. (D) Immunoblot of H3K27me3, H2AK119Ub, H3K9me3, H3K4me3, and H3K27ac levels in histones from scramble and *Pcl3* shRNA-expressing cells. (E) Pcl3-TAP resistant to *Pcl3* shRNA was reintroduced into *Pcl3* shRNA cells, immunoprecipitated, and detected with anti-FlagM2. *Suz12^Suz12TAP/+^* cells were used as a positive control. (F) Pcl3-TAP binds Suz12, Eed, and Ezh2. Lysates from scramble and *Pcl3* shRNA cells containing Pcl3-TAP were immunoprecipitated with FlagM2 and immunoblotted for Suz12, Eed, and Ezh2. (G) qRT-PCR shows partial rescue of *Pcl3* expression in *Pcl3* shRNA clones expressing *Pcl3-TAP*. Error bars indicate standard deviation. Graph represents average expression from 3–6 different clones in three experiments assayed in quadruplet. (H) Immunoblot showing restoration of H3K27me3 levels in *Pcl3* shRNA cells transduced with Pcl3-TAP. Histone H3 and α-tubulin were used as loading controls. All westerns and immunoprecipitations were performed three or more times with 2–6 clones.

PRC2 accessory proteins, such as Aebp2, Pcl1, Pcl2 and Jarid2, can either promote or inhibit PRC2 function [35]–[41], [47], [48], [50], [52]. To elucidate whether Pcl3 regulates PRC2 function, we measured H3K27me3 levels in cells with diminished Pcl3. *Pcl3* shRNA-treated ESCs and EBs showed approximately 80% depletion of H3K27me3 levels compared to scramble shRNA controls, indicating that Pcl3 promotes PRC2 function (Figure 3A and Figure S3A). To confirm this finding, we transfected ESCs with either *Suz12* or *Pcl3* siRNAs and assessed H3K27me3 levels [56], [57]. qRT-PCR and immunoblot indicated that transfection of *Suz12* siRNAs diminished *Suz12* expression in a dose-dependent fashion (Figure S3B–S3C). Similarly, transfection of *Pcl3* siRNAs resulted in decreased *Pcl3* expression and markedly reduced H3K27me3, further demonstrating that *Pcl3* promotes the formation of H3K27me3 (Figure S3D and Figure 3B). As *Pcl3* knockdown decreased H3K27me3, we assessed whether increased Pcl3 resulted in a concomitant increase in H3K27me3. Indeed, overexpression of *Pcl3* in ESCs enhanced H3K27me3 levels (Figure S2D–S2E and Figure 3C). Thus Pcl3 promotes trimethylation of H3K27.

Besides H3K27me3, histone modifications such as ubiquitination of lysine 119 of histone H2A (H2AK119Ub) and trimethylation of lysine 9 of histone H3 (H3K9me3) are involved in gene repression [58]–[60]. In addition, H3K27me3 is inversely correlated with acetylation on lysine 27 (H3K27ac) and marks bivalent domains with H3K4me3 [11], [61]. To assess whether Pcl3 participates in histone modifications apart from H3K27me3, we analyzed histones from scramble and *Pcl3* shRNA-treated cells for H2AK119Ub, H3K9me3, H3K4me3, and H3K27ac. Levels of H2AK119Ub, H3K9me3, H3K4me3, and H3K27ac were all comparable between the control and *Pcl3* knockdown ESCs, indicating that Pcl3 is not involved in global histone modification, but has a specific role in generating H3K27me3 (Figure 3D).

To confirm that *Pcl3* shRNA-associated phenotypes were due to decreased *Pcl3* levels, we created a construct encoding a TAP-tagged Pcl3 not recognized by the *Pcl3* shRNAs. Pcl3-TAP was detected by immunoblot and immunofluorescence and was able to bind core PRC2 components as assessed by co-immunoprecipitation, indicating that Pcl3-TAP functions similarly to wild type Pcl3 (Figure 3E-3F and Figure S3E). *Pcl3* expression in *Pcl3-TAP*-expressing ESCs was nearly 60% of endogenous levels as measured by qRT-PCR (Figure 3G). Re-expression of *Pcl3-TAP* increased H3K27me3 levels in *Pcl3* knockdown ESCs (Figure 3H). These data indicate that Pcl3 promotes the formation of H3K27me3 by PRC2.

### Pcl3 promotes PRC2 binding and function at a subset of PRC2 targets

To elucidate which genes depend upon Pcl3 for H3K27me3 formation and to investigate whether decreased H3K27me3 is a result of Pcl3-dependent PRC2 binding, we performed ChIP-seq for H3K27me3 and Suz12 in *Suz12^Suz12TAP/+^* cells expressing either *Pcl3* or control shRNA (GSE28325). Reads were aligned to the mouse reference genome (NCBI Build 37, MM9) using bowtie, and only uniquely aligned reads were considered for further analysis (Figure S4A–S4B) [62].

Suz12 ChIP-seq was performed using the Flag tag of Suz12-TAP. To validate this approach, we compared ChIP-seq of Suz12-TAP using anti-Flag antibodies with ChIP-seq using anti-Suz12 antibodies in *Suz12^Suz12TAP/+^* cells and found they were equivalent. We, thus, used FlagM2 to detect Suz12 binding in all the following experiments. ChIP-seq with FlagM2 antibodies detected 95% of previously reported Suz12 binding sites, indicating that this approach accurately measured Suz12-TAP localization in the genome (Figure S4C) [63], [64]. In addition, we identified a number of novel Suz12 binding sites, typified by less significant Suz12 ChIP-seq peaks (Figure S4C). These additional sites may reflect weak Suz12 binding or may be an artifact of increased Suz12 levels.

**Figure 4:**
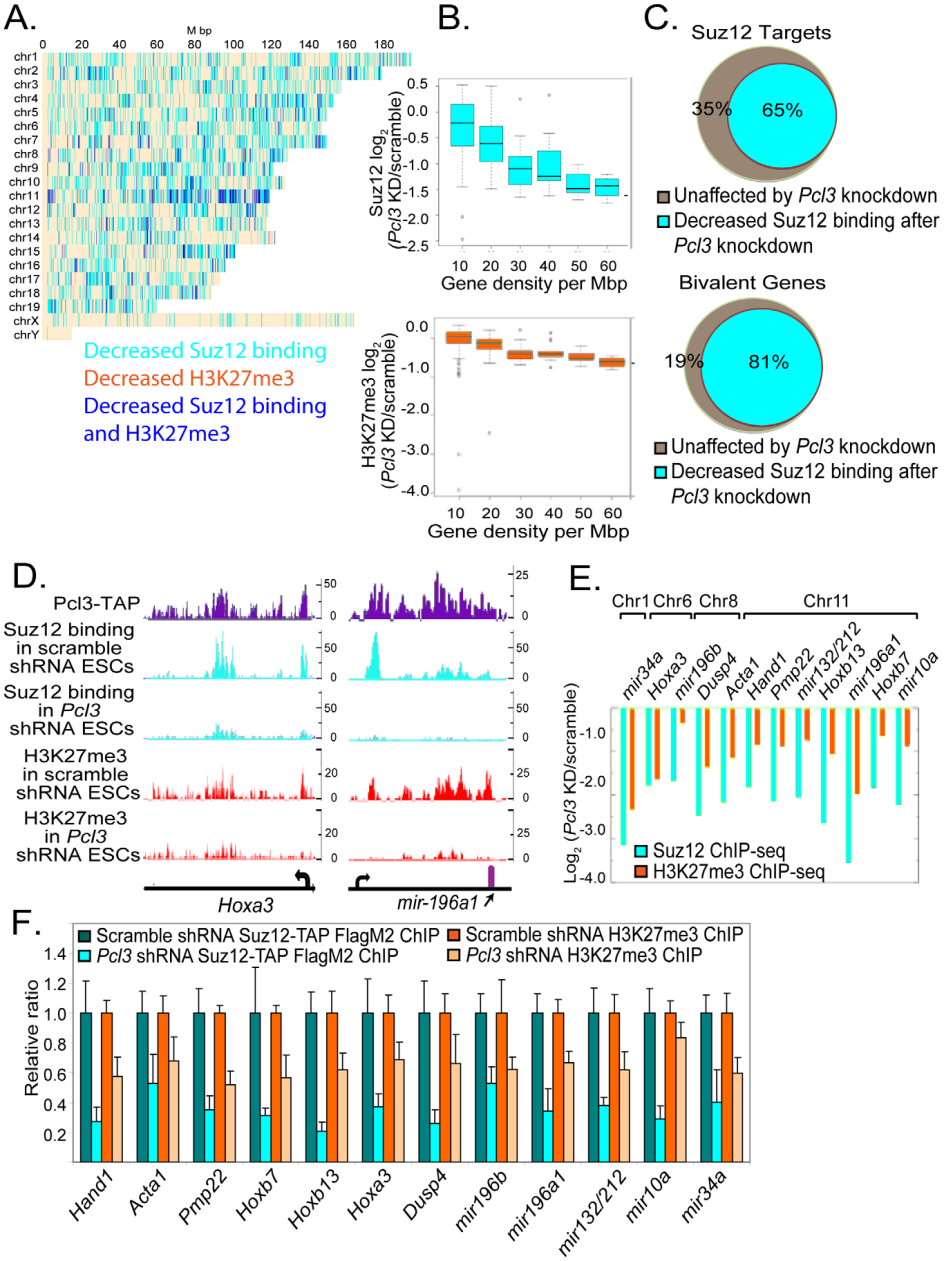
Pcl3 affects Suz12 binding at a subset of PRC2 targets. (A) Graph depicts chromosome-wise distributions of decreased Suz12 and H3K27me3 ChIP-seq reads upon *Pcl3* knockdown (*Pcl3* KD) relative to scramble control. Many regions display both Suz12 and H3K27me3 depletion (blue). Independently decreased Suz12 occupancy and H3K27me3 are marked in cyan and orange, respectively. ChIP-seq peaks were called at a Skellam distribution p-value cutoff of 10^−7^. Fisher test p-value was 3.2×10^−41^ for the overlap of H3K27me3 with Suz12 sites. (B) Reduced Suz12 and H3K27me3 ChIP-seq read density upon *Pcl3* knockdown is correlated with gene density. Pearson correlation is 0.40 for Suz12 and 0.26 for H3K27me3. (C) 65% of PRC2 targets show decreased Suz12 binding upon *Pcl3* knockdown, particularly bivalent genes (Fisher test *p* = 4.9×10^−250^). (D) Binding of Pcl3 and Suz12, and histone mark profiles of H3K27me3 at the *Hoxa3* gene and in the region surrounding *mir-196a1*. Units are number of reads/250 bp. (E) Log fold-changes in Suz12 and H3K27me3 ChIP-seq read density at specific genes in *Pcl*3 knockdown cells compared to scramble control. (F) Levels of Suz12 binding and H3K27me3 in *Suz12^Suz12TAP/+^* cells expressing either scramble or *Pcl3* shRNA assessed by FlagM2 or H3K27me3 ChIP-qRT-PCR. ChIP-qRT-PCR experiments were performed at 3–4 times and assayed in quadruplicate. Error bars indicate standard deviation. Decreases are statistically significant, *p*<0.005.

Comparison of H3K27me3 ChIP-seq in scramble and *Pcl3*-shRNA treated ESCs revealed a widespread decrease in H3K27me3 levels in *Pcl3* shRNA ESCs (Figure 4A). The decrease in H3K27me3 as assessed by ChIP-seq was similar to, but not as profound as was detected biochemically. This may be attributable to the inability of ChIP-seq to detect H3K27me3 in regions of highly repetitive sequence, areas that are largely transcriptionally silent. At approximately 85% of sites showing decreased H3K27me3, *Pcl3* knockdown cells also displayed reduced Suz12 binding compared to scramble controls, thus correlating decreased Suz12 occupation with decreased PRC2 function (Figure 4A). Indeed, *Pcl3* knockdown significantly reduced Suz12 binding at approximately 65% of PRC2 targets (Figure 4A, 4C). Sites with decreased Suz12 binding outnumbered those with reduced H3K27me3, indicating that *Pcl3* knockdown preferentially affected Suz12 (Figure 4A).

The regions with the most decreased Suz12 occupation following *Pcl3* knockdown were among the most significant Suz12 ChIP-seq peaks in control cells (Figure S4D). In contrast, less significant Suz12 peaks were more likely to be unaffected by *Pcl3* knockdown (Figure S4D). The finding that the role of Pcl3 is most pronounced on the most significant Suz12 binding sites is consistent with a role for Pcl3 in mediating the recruitment of additional Suz12 to regions of low level Suz12 binding. Alternatively, changes in Suz12 binding may be more difficult to detect at these less significant peaks.

Knockdown of *Pcl3* diminished Suz12 binding to chromatin particularly in areas of high gene density (Figure 4B). Accordingly, chromosome 11, the chromosome with the highest gene density, showed the most profound decrease in Suz12 binding and H3K27me3 upon *Pcl3* knockdown (Figure 4A and Figure S4E). Consistent with PRC2 binding at repressed genes, sites with decreased Suz12 binding in *Pcl3* shRNA-treated ESCs were inversely correlated with sites bound by the activating transcription factors E2f1, c-Myc, Zfx, Klf4, and Ctcf, (Figure S4F and data not shown, Fisher test p-value = 9.5×10^−53^, 3.7×10^−15^, 1.2×10^−13^, 4.0×10^−6^, 3.6×10^−6^, respectively) [63]. This is in agreement with previous reports demonstrating nearly mutual exclusion of Suz12 binding with regions bound by this group of transcription factors [63].

As mentioned, Suz12 and H3K27me3 are present at bivalent genes, genes with both active (H3K4me3) and repressive (H3K27me3) histone marks in ESCs [11], [65]. We found that more than 80% of bivalent genes showed decreased Suz12 binding upon *Pcl3* depletion (Figure 4C) [11], [65]. Among bivalent genes are many developmental genes and microRNAs [64], [66]–[70]. Using ChIP-qRT-PCR, we confirmed that Pcl3 is important for deployment of PRC2 at some of these developmental gene targets and microRNAs including *Hoxb7*, *Hoxb13*, *Hoxa3*, *Dusp4*, *Hand1*, *mir-196b*, *mir-196a1*, *mir-10a*, *mir-132/mir-212*, and *mir-34a* (Figure 4D–4F) [66], [67], [69], [71]–[74]. Suz12 ChIP-qRT-PCR also confirmed that some Suz12 targets (e.g., *Tle3*, *Tns1*, and *Tmem151a*) are unaffected by Pcl3 (Figure S4G). These data further demonstrate that Pcl3 promotes PRC2 binding and mediates PRC2 activity at a specific subset of PRC2 targets.

As H3K4me3 histone marks are also present at bivalent genes, we extended our ChIP-qRT-PCR analysis to H3K4me3 to assess whether *Pcl3* depletion affects H3K4me3 formation. At genes marked by H3K4me3 but not H3K27me3 (i.e., *Eaf1*, *Ash21*, *Dcaf8*, *Ift140*, *Glrx5*), H3K4me3 levels were unchanged by *Pcl3* depletion, consistent with our finding that global H3K4me3 levels are unchanged in *Pcl3* shRNA ESCs (Figure S4H and Figure 3D). H3K4me3 levels at approximately half of the bivalent genes assayed showed modestly increased H3K4me3 levels (i.e., *Acta1*, *Hoxa3*, *Ebf2*, *mir196b*, *Hand1*, *Dusp4*, *Pmp22*), while other genes were unchanged (i.e., *mir196a1*, *Hoxb7*, *Matb2*, *Pcdh7*, *Otx2*, *Hoxb13*) (Figure S4H). Thus, *Pcl3* knockdown affects H3K4me3 levels specifically at a subset of bivalent genes.

### Pcl3 co-localizes with Suz12 at many PRC2 targets to promote H3K27me3

**Figure 5:**
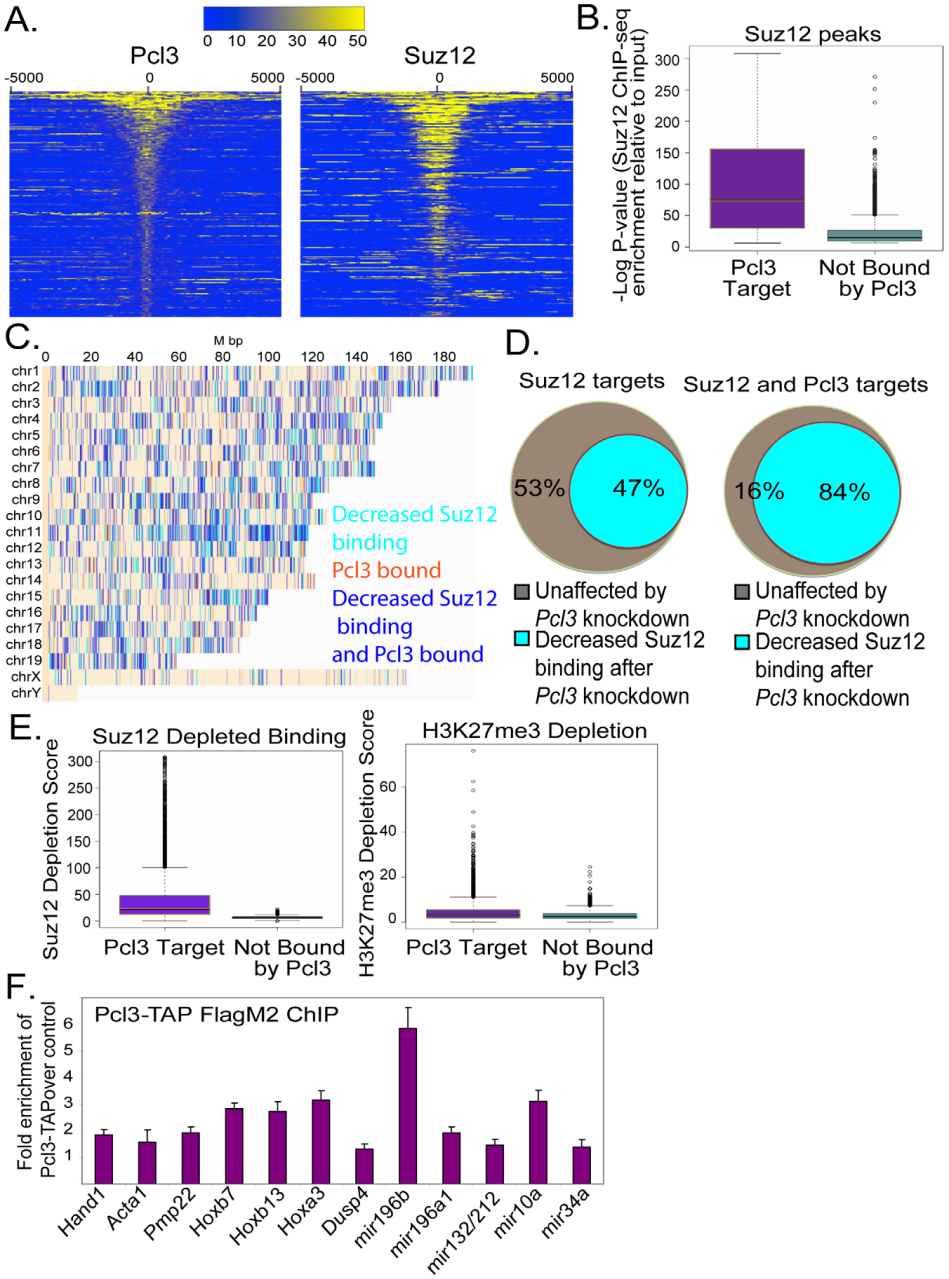
Pcl3 localizes to PRC2 targets. (A) Heatmaps showing Pcl3 and Suz12 ChIP-seq read density in counts per 100 bp around Pcl3 peak centers. Approximately 44% of Suz12 targets are bound by Pcl3-TAP. Each row corresponds to a Pcl3 ChIP-seq peak with rows ranked by Pcl3 peak significance (assessed by the Skellam distribution p-values). (B) Graph of −log p-values indicating that the most significant Suz12 ChIP-seq peaks overlap with Pcl3, while regions containing less significant Suz12 ChIP-seq peaks are not bound by Pcl3. Wilcoxon p-value<1e^−306^ for the difference in the distribution of ChIP-seq −log p-values. (C) Pcl3 co-localizes with Suz12 depletion sites (blue). Regions containing only Suz12 depletion or only Pcl3 binding are indicated in cyan and orange respectively. (D) 84% of sites bound by Suz12 and Pcl3 show decreased Suz12 binding upon *Pcl3* (Fisher test p-value = 1.5×10^−306^). Two binding sites were considered to be overlapping if their peak centers were within 2 kb from each other. (E) Suz12 (Wilcoxon p-value<1e^−306^) and H3K27me3 (Wilcoxon p-value = 3.7e^−36^) co-localizing with Pcl3 show much more significant depletion compared to Suz12 and H3K27me3 targets not bound by Pcl3. Depletion score = −log p-value (read counts before/after *Pcl3* KD). (F) Genes and microRNAs bound by Pcl3-TAP measured by FlagM2 ChIP-qRT-PCR. The graph depicts average fold enrichment over control levels for three different clones in three different experiments assayed in quadruplet. Error bars indicate standard deviation. All increases are statistically significant, *p*<0.04.

As Pcl3 binds core components of PRC2 and promotes Suz12 localization to its target genes, we investigated whether Pcl3 co-localizes with Suz12 at target loci. To assess the distribution of Pcl3 binding to chromatin in ESCs, we performed ChIP-seq of Pcl3-TAP. Nearly all of the regions bound by Pcl3-TAP overlapped with Suz12 targets, suggesting that Pcl3 co-localizes at target genes as part of PRC2 (Figure 5A and S5A–S5B, Fisher test p-value = 1.5×10^−306^). Quantification revealed that Pcl3-TAP bound nearly half of PRC2 targets, indicating that Pcl3 associates with a subset of PRC2 complexes (Figure 5A). To substantiate the co-localization analysis, we assessed which Suz12 sites overlapped with Pcl3. Pcl3 and Suz12 co-localized predominantly at the most significant Suz12 binding sites (Figure 5B). Suz12 sites at which Pcl3 did not co-localize were typified by less significant Suz12 binding (Figure 5B). In addition, 80% of Pcl3 and Suz12 sites overlapped with CpG islands compared to 65% of sites bound only by Suz12, indicating that Pcl3 and Suz12 preferentially bind at CpG islands (p-value = 2.2×10^−16^) [13].

To establish whether Pcl3 may directly contribute to PRC2 binding and function, we compared regions of Pcl3 binding to areas of Pcl3-dependent Suz12 chromatin occupation and H3K27me3 formation. Upon *Pcl3* knockdown, 84% of Pcl3 binding regions showed decreased Suz12 binding, whereas less than half of regions not bound by Pcl3 showed decreased Suz12 binding (Figure 5C–5E). In addition, Pcl3 binding regions showed a near two-fold enrichment for decreased H3K27me3 upon *Pcl3* knockdown, as compared to regions not bound by Pcl3 (Figure 5E). ChIP-qRT-PCR for Pcl3 confirmed that Pcl3 bound to many of the same genes that exhibited decreased Suz12 binding and H3K27me3 upon *Pcl3* knockdown (Figure 4F and Figure 5F). These data indicate that genes bound by Pcl3 are nearly twice as likely to have reduced Suz12 binding and H3K27me3 upon *Pcl3* knockdown. Thus, Pcl3 not only co-localizes with Suz12 at many PRC2 targets, but also promotes Suz12 binding and PRC2 function.

### Pcl3 regulates gene expression at a subset of PRC2 targets

**Figure 6:**
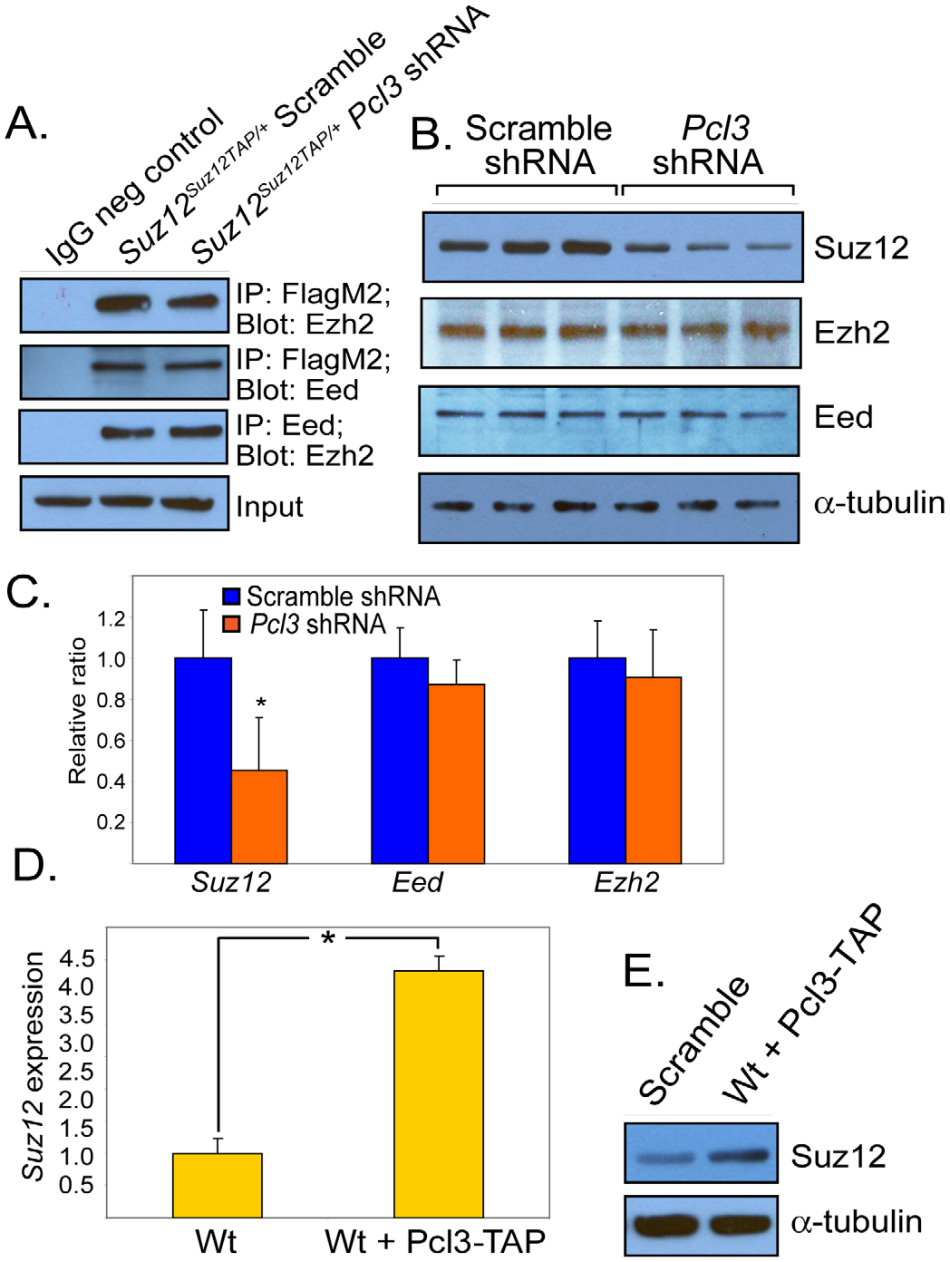
Pcl3 regulates Suz12, but not complex stability. (A) PRC2 components associate in the absence of Pcl3 based on co-immunoprecipitation. β-actin used as a loading control. (B) Protein levels of Suz12, Ezh2, and Eed in scramble and *Pcl3* shRNA clones as measured by immunoblot. Experiments were performed with 3–6 scramble and *Pcl3* shRNA clones each. (C) Expression levels of *Suz12*, *Ezh2*, and *Eed* in scramble and *Pcl3* shRNA measured by qRT-PCR. (D) *Suz12* mRNA levels measured by qRT-PCR in wild type and *Pcl3* overexpressing cells. (E) Suz12 protein levels in wild type cells and wild type cells overexpressing *Pcl3-TAP*. All immunoblot and co-immunoprecipitation experiments were performed 2–5 times. All expression analysis represents three experiments assayed in quadruplicate. Error bars represent standard deviation, and asterisk indicates statistical significance of *p*<0.005.

Inhibition of PRC2 core components and complete abrogation of H3K27me3 dramatically alter gene expression, whereas inhibition of accessory proteins such as Jarid2 result in more modest changes [22], [29], [30], [40]. To test whether Pcl3 affects gene expression, multiple scramble and *Pcl3* shRNA clones were analyzed by microarray. Nearly 130 genes displayed altered expression upon *Pcl3* knockdown. Unexpectedly, given that PRC2 functions to repress gene expression, nearly three times as many genes were down-regulated upon *Pcl3* knockdown as were up-regulated (Table S1). Notably, we identified more than half of the upregulated genes as PRC2 targets, which correlated well with depleted Suz12 binding and H3K27me3 (Figure S6B). We confirmed the microarray data by measuring gene expression by qRT-PCR for several genes (Figure 6SA).

Given that *Pcl3* knockdown increases the proportion of differentiated cells, we tested whether the presence of differentiated cells masked changes in ESC gene expression by pre-plating twice to remove differentiated cells. Microarray analysis revealed that approximately 120 genes showed differential expression in the *Pcl3* shRNA cells compared to control after removal of differentiated cells (Table S2). qRT-PCR assessment of a subset of the affected genes confirmed the microarray results (Figure S6B). Of these Pcl3-regulated genes, almost two thirds were up-regulated upon Pcl3 depletion. While these results demonstrate that Pcl3 participates in the repression of genes in ESCs, only a few of these genes also displayed decreased Suz12 binding and H3K27me3 levels (i.e., *Hand1*, *Acta1*, and *Pmp22*) (Table S2, Figure 4E–4F and Figure S6B) which may be attributable to the necessity of culturing *Pcl3* shRNA ESCs for an extended time before subjecting them to microarray analysis.

### Pcl3 regulates Suz12, but not complex stability

Loss of core PRC2 components can destabilize the complex [25], [29]. To assess whether loss of Pcl3 affects complex stability, we tested whether the complex could form in the absence of Pcl3. All components, Suz12, Ezh2, and Eed, were found by co-immunoprecipitation to bind each other in both scramble and *Pcl3* shRNA-expressing cells, suggesting that Pcl3 is not required for assembly or stabilization of the core complex (Figure 6A).

To determine whether depleting Pcl3 influences levels of PRC2 components, we assessed the quantity of PRC2 core components in *Pcl3* shRNA-treated ESCs. Interestingly, suppression of *Pcl3* significantly lowered Suz12 mRNA and protein levels without affecting levels of Eed or Ezh2, suggesting that Pcl3 regulates *Suz12* (Figure 6B–6C). Overexpressing Pcl3 caused a concomitant increase in Suz12 mRNA and protein levels (Figure 6D–6E). As *Suz12* is not a known PRC2 target and Pcl3 is not enriched at the *Suz12* locus, effects on *Suz12* expression by Pcl3 are likely indirect (Figure S6D) [7], [75], [76].

### The Tudor domain of Pcl3 is critical for its function

**Figure 7:**
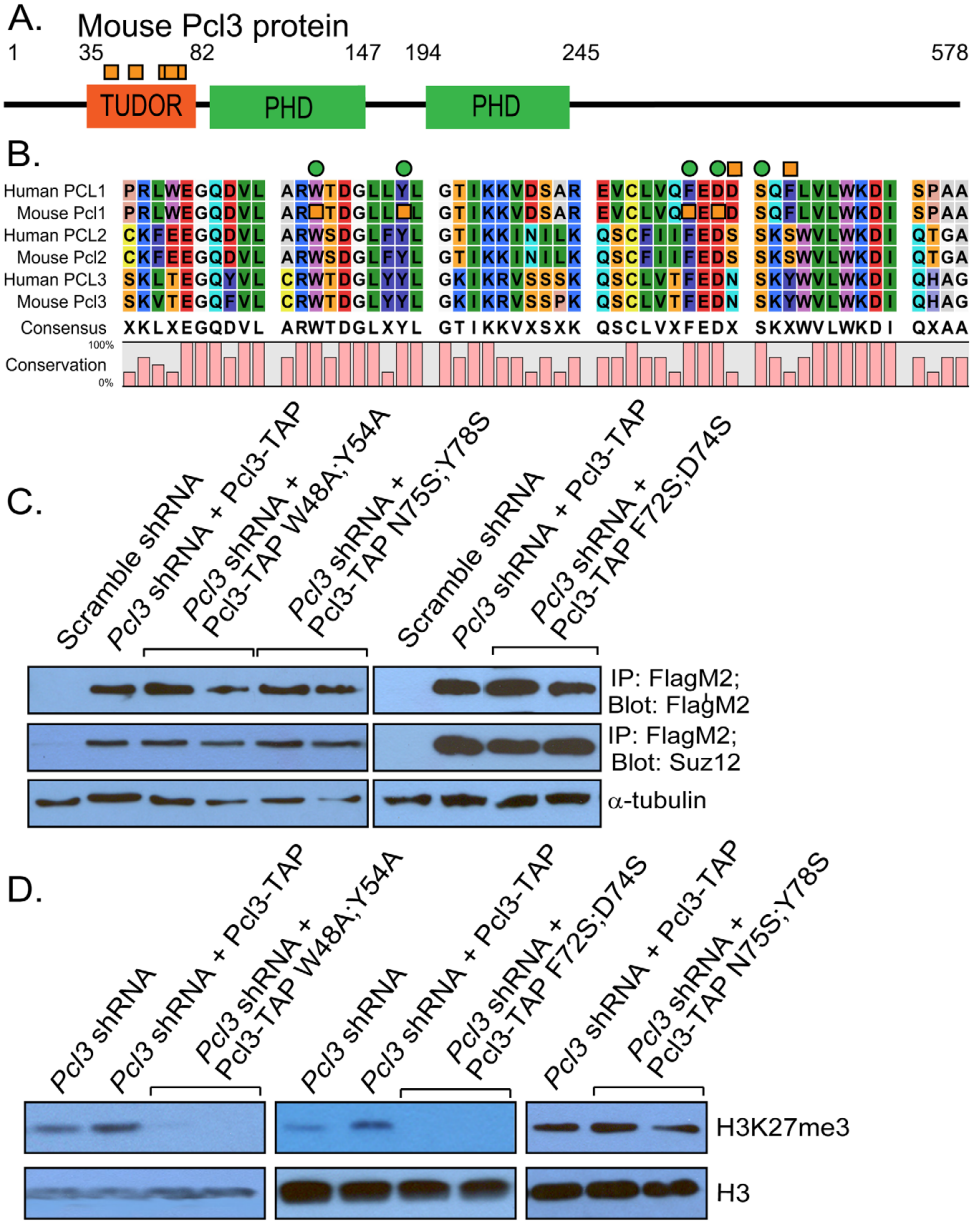
Pcl3 requires Tudor domain residues for function. (A) Schematic of Pcl3 architecture including Tudor and PHD domains. Size of domains are indicated by amino acid number, but schematic is not to scale. Orange boxes indicate mutated residues. (B) Sequence alignment of the Tudor domain of human and mouse Polycomb-like homologues. Bottom graph indicates the degree of conservation at each residue. Green circles indicate putative histone binding sites. Orange squares indicate mutated residues. (C) Mutant Pcl3-TAP protein is detected at similar levels to wild type Pcl3-TAP by immunoprecipitating and probing with anti-FlagM2. Mutant Pcl3-TAP can bind Suz12 as well as wild type Pcl3-TAP by co-immunoprecipitation. (D) Pcl3-TAP W48A;Y48A and Pcl3-TAP F72S;D74S do not support H3K27me3 whereas Pcl3-TAP N75S;Y78S does. Immunoblot displaying H3K27me3 levels in histones purified from cells expressing *Pcl3* shRNA, *Pcl3* shRNA with Pcl3-TAP, and *Pcl3* shRNA with mutant Pcl3-TAP. Histone H3 and α-tubulin were used as loading controls. Each lane represents a different clone. All westerns and immunoprecipitations were performed 3–4 times with at least two clones from each mutant.

Polycomb-like proteins contain three domains, an amino-terminal Tudor domain and two carboxy-terminal PHD fingers (Figure 7A). Although the carboxy PHD finger of Pcl2 promotes PRC2 recruitment, the Tudor domain and amino PHD finger of Pcl2 are not required for its regulation of PRC2 [41], [77]. In contrast, the Tudor domain and carboxy-terminal PHD finger of human Pcl3 are important for binding Ezh2 and self-association [53].

NMR studies indicate that the Drosophila Polycomb-like Tudor domain lacks a complete aromatic cage, needed to bind methylated lysines or arginines on histones [78]. Interestingly, a comparison of Drosophila Pcl Tudor domain sequence to those of mammalian Pcl Tudor domains suggests that mammalian Polycomb-like proteins may be able to form complete aromatic cages [78]. In particular, the conserved residues W48,Y54, F72, D74, and S76 are implicated in histone binding (Figure 7A–7B) [78]. To ascertain if these Tudor domain residues are required for Pcl3 function, we mutated two sets (W48;Y54 and F72;D74), as well as a control set of residues (N75;Y78) within Pcl3-TAP (Figure 7A–7B) [78]. These mutant forms of Pcl3 were expressed in *Pcl3* knockdown ESCs to see if they could rescue H3K27me3 levels as well as wild type Pcl3-TAP (Figure 7C). Similar to wild type Pcl3-TAP, Pcl3-TAP N75S;Y78S was able to promote H3K27me3 formation (Figure 7D). In contrast, Pcl3-TAP W48A;Y54A and Pcl3-TAP F72S;D74S did not restore H3K27me3 formation (Figure 7D). Thus, W48;Y54 and F72;D74, two pairs of residues implicated in histone binding, are necessary for Pcl3 function, while the control N75;Y78 pair is dispensable.

To discern whether these Tudor domain residues are necessary for Pcl3 incorporation into PRC2, we assessed whether Suz12 co-immunoprecipitated with Pcl3-TAP W48A;Y54A, Pcl3-TAP F72S;D74S, and Pcl3-TAP W48A;Y54A. All mutant forms of Pcl3 associated with Suz12, indicating that these residues are not required for binding PRC2 (Figure 7C). Thus, W48;Y54 and F72;D74 are essential for Pcl3 promotion of H3K27me3 formation, but not for Pcl3 incorporation into PRC2.

### Pcl3 and Pcl2 are part of distinct PRC2 complexes with overlapping targets

**Figure 8:**
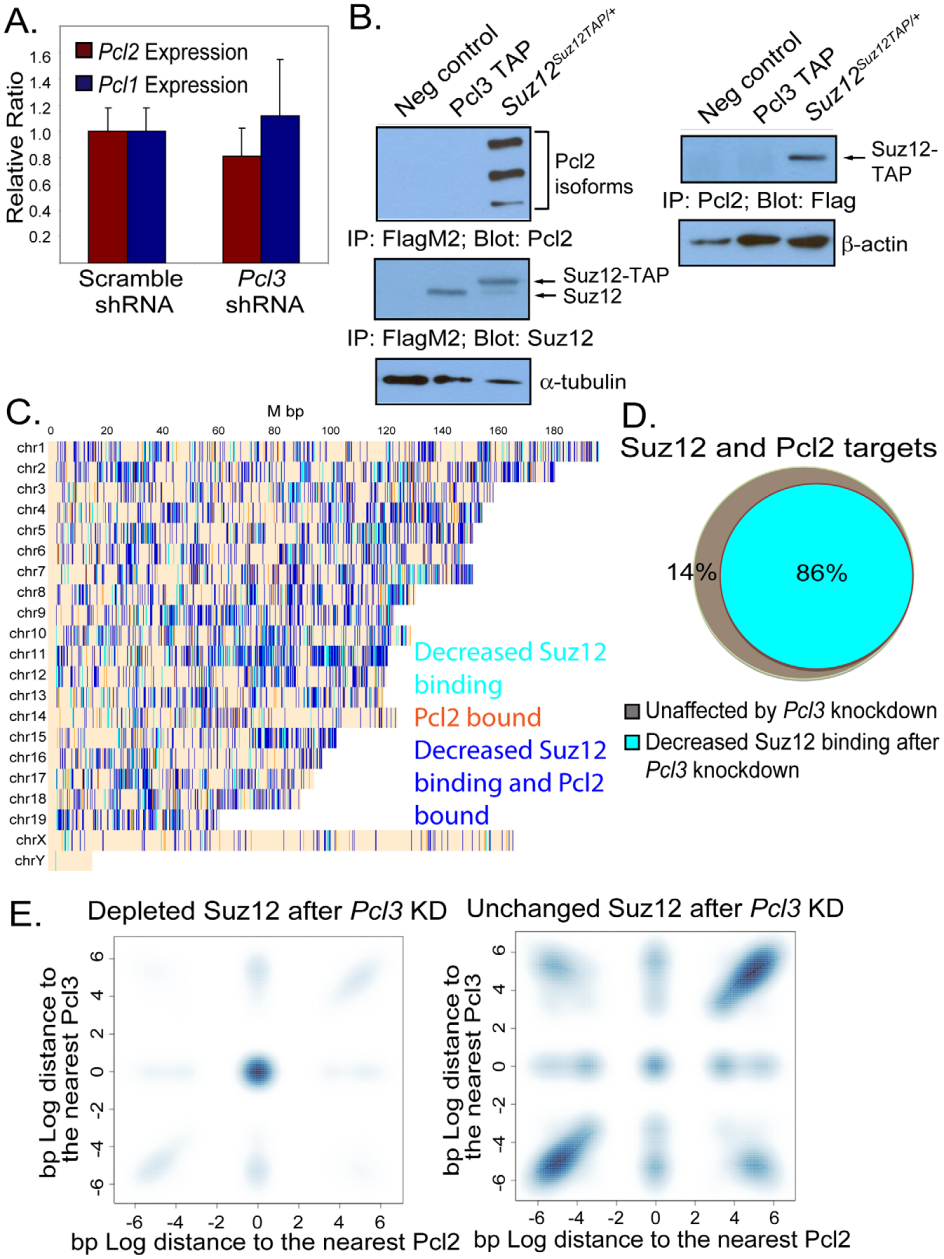
Pcl3 and Pcl2 participate in separate PRC2 complexes but overlap at many PRC2 binding sites. (A) *Pcl2* and *Pcl1* mRNA levels are not significantly different between scramble and *Pcl3* shRNA cells. Graph represents average expression from three different experiments in 2–6 different clones and assayed in quadruplet. Error bars indicate standard deviation. (B) Co-immunoprecipitation showing that immunoprecipitated Suz12-TAP associates with Pcl2 whereas immunoprecipitated Pcl3-TAP does not. Pcl3-TAP does bind Suz12 as seen in the lower blot. α-tubulin was used as a loading control. Reciprocal co-immunoprecipitations showing a Pcl2-specific association with Suz12-TAP but not an association between Pcl2 and Pcl3-TAP. β-actin was used as a loading control. Each blot is representative of three different experiments. (C) Graph depicts chromosome-wise distributions of Pcl2 co-localization with sites depleted of Suz12 following *Pcl3* knockdown (blue). Regions containing only Suz12 depletion and only Pcl2 binding are indicated in cyan and orange respectively. (D) Decreased Suz12 binding upon *Pcl3* knockdown occurs at 86% of targets bound by Suz12 and Pcl2 (Fisher test p-value<p<10^−300^). Of sites bound by Pcl2 and Pcl3, 95% show decreased Suz12 binding upon *Pcl3* knockdown. (E) Pcl2 and Pcl3 binding sites co-localize with regions of Suz12 depletion upon *Pcl3* knockdown (top), whereas Pcl2 and Pcl3 binding sites reside far from unaffected Suz12 binding sites (bottom). The figure shows the joint probability density of the nearest Pcl2 and Pcl3 locations relative to Suz12. Darker color represents higher probability density. Bivariate normal kernel density estimates were obtained by using the smoothScatter function in R. The distance coordinates were transformed to base pairs sign (Pcl2−Suz12) log_10_ |Suz12−Pcl2|, and similarly for Pcl3.

While Pcl1 is minimally transcribed in ESCs, Pcl2 is highly expressed and binds a subset of PRC2 target genes [41]. To assess whether depletion of Pcl3 affects Pcl1 or Pcl2, we measured *Pcl1* and *Pcl2* expression in scramble and *Pcl3* knockdown ESCs. Pcl1 was expressed at extremely low levels but showed no difference upon *Pcl3* knockdown (Figure 8A). Likewise, we found that *Pcl2* levels were similar between scramble and *Pcl3* shRNA-treated ESCs (Figure 8A).

Although Pcl2 and Pcl3 are both expressed in ESCs, it is unclear if they participate in same complex or regulate the same genes. To assess whether Pcl3 and Pcl2 incorporate into the same complex or exist in distinct PRC2 complexes, we tested Pcl3 and Pcl2 association by co-immunoprecipitation. Flag immunoprecipitation confirmed that Pcl2 and Pcl3 both bind Suz12 (Figure 8B). However, Pcl3-TAP did not immunoprecipitate Pcl2, suggesting that Pcl2 and Pcl3 do not associate within the same PRC2 complex (Figure 8B). To substantiate these data, we performed the reciprocal immunoprecipitation and found that Pcl2 immunoprecipitated Suz12-TAP but not Pcl3-TAP. These findings indicate that Pcl2 and Pcl3 both interact with the core PRC2 complex, but not with each other, suggesting that Pcl2 and Pcl3 participate in distinct PRC2 complexes.

To elucidate whether Pcl3 and Pcl2 bind distinct or overlapping sets of targets, we compared our Pcl3 ChIP-seq data with previously generated Pcl2 ChIP-seq data and found that Pcl3 and Pcl2 binding overlaps at 60–70% of sites [41]. This extensive overlap raises the possibility that many genes may be regulated by both Pcl3 and Pcl2.

Like Pcl3, Pcl2 can promote PRC2 activity at some loci and in some cell types [41], [47], [77], [79]. To test whether Pcl2 can compensate for Pcl3 function, we analyzed whether Suz12 binding at Pcl2 targets was affected by Pcl3 depletion. Of sites bound by both Suz12 and Pcl2, 86% showed decreased Suz12 binding upon Pcl3 depletion (Figure 8C–8D), suggesting that Pcl2 cannot compensate for Pcl3 function at Pcl2 sites.

Unlike Pcl3, Pcl2 can either inhibit or promote PRC2 function [41], [47], [77], [79]. To further investigate the functional relationship between Pcl2 and Pcl3, we assessed Suz12 binding and H3K27me3 at regions bound by both Pcl2 and Pcl3. If Pcl2 and Pcl3 act redundantly to promote Suz12 binding, sites bound by both would not show significant decreases in Suz12 binding upon *Pcl3* knockdown as Pcl2 would be functional at these sites. Conversely, if Pcl2 hinders PRC2 binding while Pcl3 promotes PRC2 binding, Suz12 binding at shared Pcl2 and Pcl3 sites would be more dramatically reduced upon *Pcl3* knockdown, as Pcl3 would no longer be able to counterbalance Pcl2 function. Among regions bound by Suz12, Pcl2, and Pcl3, 95% showed Suz12 depletion upon *Pcl3* knockdown (Figure 8C–8D). Moreover, Pcl3-dependent Suz12 sites often co-localized with Pcl2 and Pcl3 binding, whereas Pcl3-independent Suz12 sites tended to be more distant from Pcl2 and Pcl3 binding sites (Figure 8E). In addition, 88% of Pcl2 and Pcl3 shared sites showed diminished H3K27me3. Thus, these data suggest that not only does Pcl2 not compensate for Pcl3, but also that Pcl2 may hinder Suz12 binding and H3K27me3 at Pcl2 and Pcl3 shared binding sites.

### Pcl2 and Pcl3 co-localize with Suz12 at CpG islands

**Figure 9:**
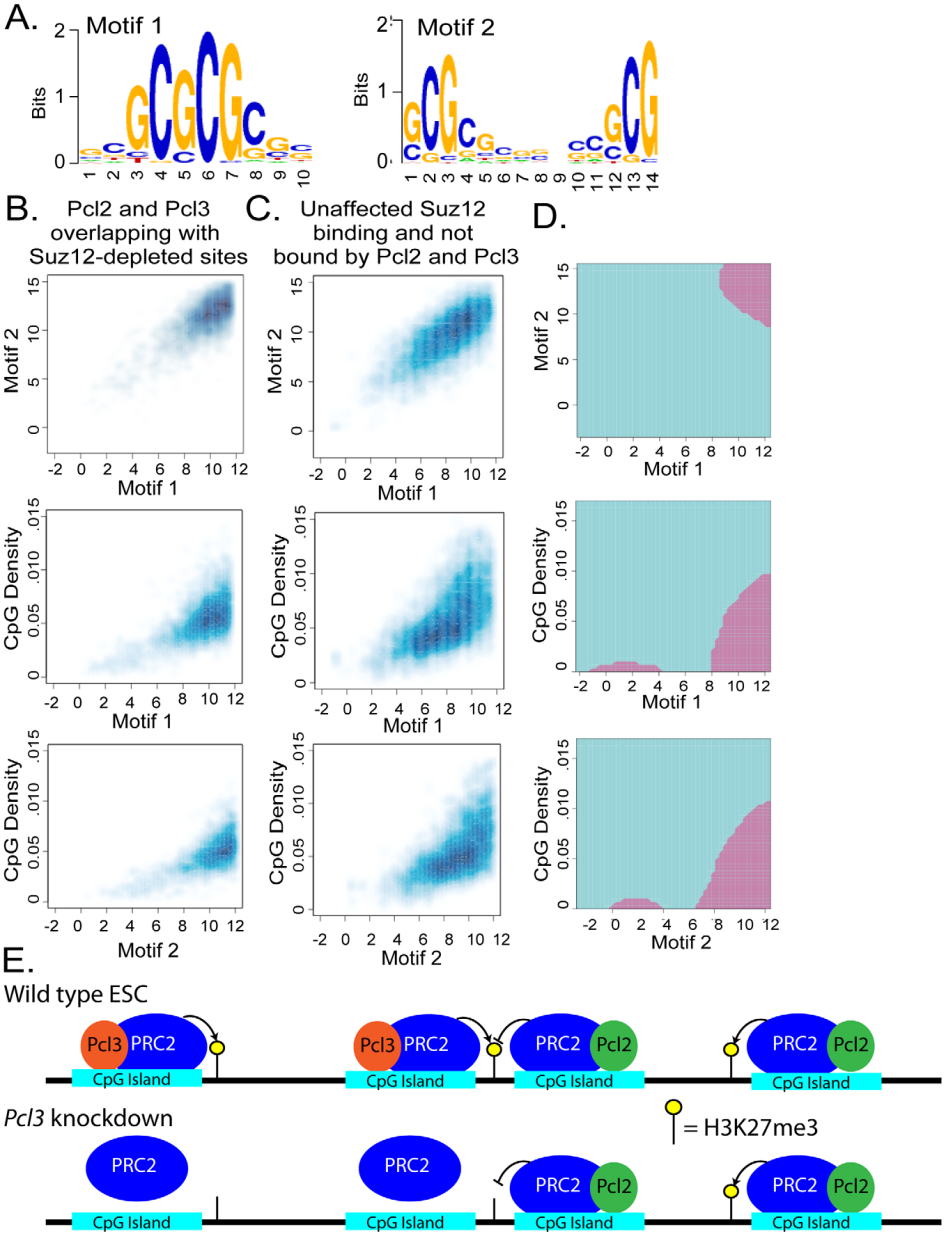
Pcl2 and Pcl3 localize to CpG islands. (A) The 500 bp central regions of Pcl3 ChIP-seq peaks were scanned for enriched motifs by using a 9th order Markov background dependence model [86]. Two examples of 10- and 14-mer enriched motifs are shown. (B–C) Smoothed scatter plots of maximum position specific-scoring matrix (PSSM) scores for the two motifs and CpG density are shown for (B) Suz12 binding sites depleted upon *Pcl3* knockdown overlapping with Pcl2 and Pcl3 and (C) Suz12 binding sites unaffected upon *Pcl3* knockdown and that do not overlap with Pcl2 and Pcl3. (D) Shown are the decision boundaries of a support vector machine classifier using these three features, where the purple regions correspond to Suz12 co-localizing with Pcl2 and Pcl3. The predictor had a cross validation accuracy of 75%. (E) A model of Pcl3 and Pcl2 regulation of PRC2 binding and activity. In wild type ESCs, Pcl3 promotes PRC2 binding and H3K27me3. Pcl2 antagonizes Pcl3-mediated Suz12 binding at sites bound by both but promotes PRC2 function at sites solely regulated by Pcl2. Knockdown of *Pcl3* causes decreased PRC2 binding and H3K27me3. Pcl2 does not compensate at Pcl2 and Pcl3 targets and continues to inhibit or promote PRC2 function depending on the gene.

Polycomb repressive elements (PREs), binding sites that recruit Polycomb proteins, have been well-described in Drosophila, but few have been found in mammals [80]–[84]. Nevertheless, mammalian PRC2 is known to preferentially bind CpG islands [13], [19], [85]. Similarly, we found that 86% of Pcl2 and Pcl3 co-localization sites were found in CpG islands [13], [41], [86]. To assess whether distinct DNA sequence motifs required for Pcl2 and Pcl3 recruitment exist, we searched for sequence features that can discriminate DNA sequences in genomic regions bound by both Pcl2 and Pcl3. We scanned 500 base pair regions surrounding Pcl3 ChIP-seq peaks for prevalent DNA sequences and identified two enriched 10- and 14-mer motifs (Figure 9A). Motif 1 consisted of three consecutive G-C dinucleotides with two nucleotides on either side containing modest GC enrichment. Motif 2 contained two G-C-rich regions separated by seven nucleotides with little to no GC enrichment. Thus, motifs commonly found in Pcl2 and Pcl3 binding regions contain short GC sequences surrounded by dissimilar sequence.

To assess whether these motifs were predictive of Pcl2 and Pcl3 binding sites, Pcl3-dependent Suz12 binding sites or CpG islands, we constructed a position specific scoring matrix (PSSM) for each motif. The occurrence of motifs 1 and 2 correlated more tightly with Pcl3-dependent Suz12 binding sites that co-localize with both Pcl2 and Pcl3 binding sites than with Suz12 sites that are not associated with Pcl2 and Pcl3 binding, nor depend on Pcl3 activity (Figure 9B–9C). In addition to the maximum PSSM scores, we also computed the CpG density of each site and used these three sequence features to build a support vector machine classifier to help predict whether specific motifs would co-localize with Pcl2 and Pcl3 binding and areas of Suz12 depletion following *Pcl3* knockdown (Figure 9D). The classifier had cross-validation accuracy of 75%, indicating that CpG density, Motif 1, and Motif 2 together account for the majority of Pcl3-dependent PRC2 binding. Even though the vast majority of Pcl2 and Pcl3 binding sites were found in CpG islands, they seemed to avoid regions with the highest CpG density, concordant with the moderate GC content of the motif sequences (Figure 9B–9D). These data indicate that Pcl2 and Pcl3 may utilize specific binding motifs to recruit PRC2 to form H3K27me3 at CpG islands.

## Discussion

In an effort to understand the mechanisms that regulate PRC2, we have discovered that a PRC2 interacting protein, Polycomb-like 3, promotes ESC self-renewal and mediates PRC2 binding at a number of targets genes. In ESCs, Pcl3 promotes the expression of multiple markers of pluripotency. Consistent with a functional role for Pcl3 in PRC2, Pcl3 also promotes H3K27me3 formation. ChIP-seq analyses revealed that diminished Pcl3 decreased Suz12 binding, particularly in regions where Pcl3 co-localizes with Suz12, suggesting that one way that Pcl3 promotes PRC2 activity is by mediating PRC2 binding to chromatin. Pcl3 likely resides in a PRC2 complex distinct from that which incorporates its paralog, Pcl2, but both PRC2 complexes have overlapping targets predominantly at CpG-rich regions. Despite their overlap and homology, Pcl2 cannot compensate for Pcl3 and may inhibit PRC2 at shared genes, as regions of Pcl2 and Pcl3 co-binding show the most significant reduction in Suz12 binding upon *Pcl3* knockdown.

Pcl3 likely has a direct role in promoting PRC2 binding at select targets, as it 1) biochemically interacts with PRC2, 2) binds to genomic regions overlapping with Suz12, and 3) is predominantly required for genomic Suz12 binding at Pcl3 binding sites. This co-occurrence and dependence of PRC2 on Pcl3 suggests that the biochemical interaction of Pcl3 with PRC2 underlies its role in PRC2 binding. In addition to this direct role for Pcl3 in PRC2 binding to chromatin, the reduction in Suz12 binding upon *Pcl3* knockdown may also be partially attributable to decreased Suz12 levels. Suz12 is not known to be regulated by PRC2 and Pcl3 does not bind the *Suz12* locus, indicating that this regulation of Suz12 by Pcl3 is likely to be indirect.

Many bivalent genes are involved in regulating ESC differentiation [8]–[15]. However, we observed that Pcl3 promotes ESC self-renewal, but is not required for differentiation into cell types of all germ layers. As we have found that Pcl3 promotes H3K27me3 at a subset of bivalent genes, it is possible that Pcl3 predominantly represses bivalent genes involved not in cell fate decisions in differentiating cells, but in genes that promote differentiation itself. Thus, Pcl3 depletion would derepress genes that promote differentiation, leading to specific defects in self-renewal. Conversely, Pcl3 overexpression may lead to increased self-renewal by repressing genes involved in differentiation that inhibit the expression of self-renewal genes such as *Nanog*.

Consistent with this idea, we found that inhibiting Pcl3 function increased H3K4me3 levels and expression at some but not all bivalent genes. Pcl3-dependent H3K4me3 may regulate be the genes that promote differentiation upon Pcl3 inhibition. Thus, diminishment of Pcl3 does not derepress all PRC2 targets, but instead may derepress a specific subset that restrain ESC differentiation.

PRC2 is recruited to chromatin by multiple mechanisms including interactions with CpG islands, non-coding RNAs, and histone binding proteins [36], [38], 39,87–89. Our analysis of Suz12 and Pcl3 binding sites revealed that they associate with CpG-enriched regions, particularly two CpG-rich DNA sequence motifs. These motifs contained GC rich regions surrounded by areas with minimal GC content, indicating that Pcl proteins do not associate with the most GC-rich regions, but rather with regions of moderate GC content and containing motifs 1 or 2. Our support vector machine classifier supported these findings by showing that Pcl2 and Pcl3 binding sites that overlap with Pcl3-dependent Suz12 bound sites localize in areas with moderate CpG density. The finding that Pcl proteins associate with CpG islands is consistent with prior observations that PRC2 binds to CpG islands and demonstrates that Pcl3 may participate in PRC2 recruitment to CpG islands [13], [19], [85].

Pcl proteins contain multiple domains by which they can associate with chromatin, including a Tudor domain and two PHD fingers implicated in recognizing methylated or unmethylated histone lysines or arginines [90]–[92]. Recent work has implicated several residues of the Pcl Tudor domain in histone binding [78]. Mutating two sets of these Pcl3 Tudor domain residues revealed that they are essential for Pcl3-dependent PRC2 activity. As these mutant forms were able to incorporate into PRC2, they may be acting as dominant negatives and poisoning the complex or as loss-of-function mutants by creating less-functional complexes. Thus, these mutants may perturb PRC2 function through inhibition of complex recruitment to histones or by decreasing PRC2 methyltransferase activity.

Two other paralogs of Pcl exist in mammals. Pcl1 is expressed minimally in ESCs, and Pcl2 has been implicated in either promoting or inhibiting PRC2 in a gene- and cell type-dependent manner [41]. In ESCs, Pcl2 restrains PRC2 activity globally, and in MEFs, Pcl2 inhibits PRC2 recruitment to certain loci [41], [47], [77]. In an effort to understand the relationship between these paralogs, we found that Pcl2 and Pcl3 do not associate and likely participate in separate PRC2 complexes, which both bind a subset of PRC2 targets. If Pcl2 and Pcl3 both promoted PRC2 binding and activity at these targets, we might expect Pcl2 to compensate in Suz12 binding and H3K27me3 upon loss of Pcl3. Instead, we observed the greatest dependence of Suz12 binding on Pcl3 at regions also bound by Pcl2, suggesting that Pcl2 may inhibit PRC2 binding at these genes (Figure 9E).

It is unlikely that Pcl2 requires Pcl3 for activity as knockdown of *Pcl2* and *Pcl3* in ESCs results in opposite phenotypes, further suggesting that these two paralogs have distinct and opposing functions. For example, Pcl2 destabilizes Ezh2, whereas Pcl3 enhances Suz12 protein levels without affecting Ezh2 [41]. Moreover, Pcl2 and Pcl3 have opposite effects on ESC self-renewal and differentiation. Pcl2 inhibits expression of *Oct4*, *Sox2*, and *Nanog* and promotes ESC differentiation [41]. In contrast, Pcl3 promotes *Oct4* and *Nanog* levels and ESC self-renewal. Finally, whereas the Tudor domain of Pcl2 is dispensable for its function, the Pcl3 Tudor domain is essential for mediating PRC2 activity [41].

Despite its global requirement to restrain H3K27me3 levels in ESCs, Pcl2 can promote H3K27me3 and PRC2 binding at certain sites [41], [47]. Pcl2 and Pcl3 overlap at approximately 65% of sites, indicating that each binds separately at a third of their sites. While our data indicates that Pcl3 and Pcl2 function oppositely at Pcl2 and Pcl3 targets, one possibility is that Pcl2 promotes PRC2 activity at regions devoid of Pcl3 (Figure 9E) [41].

Based on these data, we propose that Pcl2 and Pcl3 have opposing functions on PRC2 binding and activity at sites they co-regulate (Figure 9E). At sites that Pcl2 and Pcl3 individually regulate, each may both promote PRC2 binding. Upon loss of Pcl3, sites dependent upon Pcl3 for promoting PRC2 function lose H3K27me3, and this loss may be exacerbated by negative regulation of PRC2 by Pcl2 at sites that Pcl2 and Pcl3 regulate together (Figure 9E). Thus, Pcl2 and Pcl3 may function antagonistically at shared sites, but promote H3K27me3 at sites regulated by a single Pcl (Figure 9E). This model predicts that the minority of sites at which Pcl2 promotes H3K27me3 levels will be regions not bound by Pcl3. The model does not resolve how Pcl2 could promote PRC2 activity at regions where Pcl3 does not bind, but could inhibit PRC2 activity at shared sites upon Pcl3 knockdown. One possibility is that regions of Pcl2 and Pcl3 co-occupancy are characterized by an inhibitory PRC2 activity independent of Pcl2.

Both bivalency and gene repression at CpG promoters are dependent on PRC2 activity. In ESCs, CpG promoters may be poised for activation, reflected by their ability to bind RNAPII even when inactive [93]. Pcl3-mediated deployment of PRC2 at CpG islands may be one mechanism by which these poised CpG promoters are kept in check in ESCs. When cell type-specific promoters are being repressed during development, Pcl3 may promote PRC2 binding at many of these same CpG islands in ways that recruit PRC1 to ensure more permanent transcriptional silencing.

Understanding how Pcl proteins regulate PRC2 at specific genes may also be critical to elucidating gene misregulation during cancer. Many cancers show a global silencing at gene promoters, particularly at CpG islands [94], [95]. Ezh2 is one of the most commonly misregulated genes in cancer, and recent work has shown that Pcl3 is misregulated in diverse cancers as well [34], [96]. Similar to our findings in ESCs, Pcl3 upregulation in cancer cells may promote PRC2 recruitment, inhibiting the expression of genes that inhibit self-renewal, and leading to inappropriate cell proliferation. Thus, studying the role of Pcl3 in PRC2 function in ESCs may provide insight into how Pcl3 up-regulation returns cancer cells to transcriptional states and self-renewal capacities similar to those of ESCs.

## Materials and Methods

### Tissue culture

Mouse E14 embryonic stem cells were grown as described previously [54], [97]. Bay Genomics gene trap line XG122 was used for the establishment of *Suz12^Suz12TAP/+^* ESCs [54].

### Lentiviral infection

ESCs were trypsinized and 1 ml of cells at 10^5^/ml were plated into one well of a 6-well plate along with 15 µl of concentrated lentivirus (Open Biosystems RMM4534 and Sigma SHC002). The plate was shaken every ten minutes for one hour and then incubated overnight at 37°C. Media was changed the next day and selection was started (2 µg/ml puromycin, 50 µg/ml zeocin). Cells were selected for six days, clones were picked, and knockdown was assessed. Five different *Pcl3* shRNA lentiviral constructs were tested and all gave similar results.

### Transfection

Cells were transfected using Lipofectamine 2000 (Invitrogen 11668-019). Briefly 0.5 µg of DNA was combined with 1 µl of Lipofectamine in 50 µl of Optimem and incubated for 20 min. ESCs were trypsinized and 2×10^5^ were plated into a well of a 6-well along with Lipofectamine mixture. Cells were incubated for 18 hrs followed by media replacement. For siRNAs, 300 ng siRNA and 3 µl Lipofectamine in 300 µl of Optimem was used for the Lipofectamine mixture.

### In vitro prepared siRNA pools

Libraries of siRNAs were made as previously described [56], [57].

### Purification and mass spectrometry of Suz12

Suz12-TAP and bound proteins were purified and subjected to mass spectrometry as previously described [79].

### Histone purification

Histones were purified from ESCs as previously described [98].

### Immunoprecipitation and Western immunoblot

Cells were treated as previously described [99]. For Flag immunoprecipitation, 40 µl of FlagM2 resin (Sigma A2220) was added to 800 µg protein lysate overnight. All experiments were performed three or more times. *Pcl3* knockdown experiments contained at least two scramble and *Pcl3* shRNA clones.

### Antibodies

The list of antibodies used for immunoblotting and immunofluorescence is included in Text S1.

### Immunofluorescence and staining

Cells were plated on coverslips coated with poly-lysine and Matrigel (BD Biosciences) and stained as previously described [99].

### Expression analysis

Expression was assayed as described previously [99]. Expression levels are representative of three or more experiments using 2–6 different clones and assayed in quadruplet. Statistical significance was determined using an unpaired student's *t*-test. Primers for qRT-PCR and ChIP is included in Text S1.

### DNA mutagenesis

Point mutations were created using the Agilent site-directed mutagenesis kit (Quikchange II XL Site-Directed Mutagenesis, Agilent Technologies, 200522) and Quikchange primer design application.

### Alignment

Polycomb-like paralog alignment was performed using CLC sequence viewer (CLC Bio).

### Alkaline phosphatase activity assay

Alkaline phosphatase activity was determined by staining and using StemTag Alkaline Phosphatase Activity Kit (Cell Biolabs CBA-301). ESCs were plated with ES media containing LIF and allowed to grow to 80% confluency. For staining, ESCs were fixed in 0.5% glutaraldehyde and washed three times in HBS followed by three washes in AP buffer (100 mM Tris pH 9.5, 100 mM NaCl, 50 mM MgCl_2_). NBT/BCIP substrate was added and samples were incubated at 37° for 10 minutes. Reaction was stopped with 50 mM EDTA pH5.1 in PBS, and pictures were taken. For the StemTag colorimetric assay, cells were lysed, incubated with assay substrate, and assayed for absorbance. Experiment was performed in duplicate three independent times.

### Colony formation assay

ESCs were trypsinized, counted, and plated at 100 cells/well of a 6-well. After 8 days, colonies were stained with methylene blue and quantitated. For wild type and Pcl3-overexpressing cells, ESCs were grown in 5% of normal LIF concentration. Experiment was performed in duplicate four independent times.

### Teratoma formation

ESCs were trypsinized, counted, and prepared to a concentration of 6.7×10^6^ cells/ml. 300 µl (2×10^6^) cells were injected subcutaneously into severely combined immunodeficient (SCID) mice and allowed to form teratomas for 2–3 weeks. Tumors were removed and divided for paraffin sections, frozen sections, and RNA. Teratomas were formed from two clones each of scramble and *Pcl3* shRNA ESCs.

### Microarray

Sample preparation, labeling, and array hybridizations were performed according to standard protocols from the UCSF Shared Microarray Core Facilities and Agilent Technologies (http://www.arrays.ucsf.edu and http://www.agilent.com). See Text S1 for more detailed description. The microarray was performed in 2–6 clones from scramble and *Pcl3* shRNA ESCs.

### ChIP

ChIP-seq experiments were done as previously described with 500 µg of chromatin and 5 µg of antibody [100]. ChIP-qRT-PCR experiments were performed at least two times with 10^7^ ESCs and 10 µg of antibody. Antibodies used for immunoprecipitation include anti-H3K27me3 (Upstate, 07-449), anti-Flag M2 (Sigma, 088k6018), anti-Flag M2 (Cell Signaling, 2368), anti-Suz12 (Upstate, 07-379), and anti-H3K4me3 (Millipore clone MC315 04-745) antibodies. More detailed descriptions are included in Text S1.

### ChIP-seq analysis

The data consisted of short reads from single-end 30-nucleotide sequencing. The short reads were aligned to the reference mouse genome (version MM9) by using bowtie with the parameters -n 2 -y –best -m 1 [62]. The summary of alignment results is provided in Figure S4A. Paired samples were normalized by equalizing the number of reads in the background: partial sums of order statistics of binned read counts in paired samples were computed, and the ratios of the partial sums were plotted as a function of the quantile cutoff in the sum (Figure S4B). The point where the curve deviates significantly from linearity was found by fitting a linear regression using half of the data between the 10th and 60th quantile and by finding the first point that deviates from the linear regression line by more than 3 standard deviations of the estimated error. The ratio of partial sums at this critical point was used to scale the read density in one of the samples. The significance of the difference in read counts was assessed by using the Skellam distribution as a null model. We used 400 bp running windows to scan the genome. A *p*-value cutoff of 10^−7^ was used to call significant windows, because this cutoff roughly corresponds to an adjusted *p*-value of 0.05 based on the method of Poisson clumping heuristic for approximating the probability of locally correlated rare events. The relation between the Skellam and adjusted *p*-values is given by *p*_Skellam_ = −log(1−*p*_adjusted_) E[C]/L, where L is the genome size, and E[C] the expected correlation length of Skellam *p*-values across adjacent windows. Overlapping significant windows were concatenated, and we report the new *p*-values recomputed in the joined windows. More detailed descriptions are included in Text S1.

### Gene density analysis

The MM9 mouse genome was partitioned into disjoint 1 Mbp bins. The log fold-change of normalized counts in paired ChIP-seq data with *Pcl3* knockdown or scramble was computed in each bin. Gene density was computed by counting the number of RefSeq genes in each 1 Mbp bin. Pearson correlation was computed between gene density and log fold-change across bins. In Figure 4B, we grouped the bins into units of 10; e.g., 10 denotes bins with gene density greater than or equal 0 and less than 10.

### Suz12 binding sites in miRNA promoters

To find miRNAs targeted by Suz12, we used a prior annotation of miRNA promoters [64]. The differential Suz12 binding levels shown in Figure 4D–4E were computed within Suz12 ChIP-seq peaks overlapping with the annotated promoters.

### Motif analysis

We used a greedy algorithm called LeitMotif which models the background nucleotide distribution with the 9^th^ order Markov dependence [86]. Motif lengths ranging between 6 and 15 were used for scanning. Top scoring motifs with length 10 and 14 were chosen for further analysis, because other motifs were either contained in or contained those two motifs. Sequences obtained from LeitMotif were aligned to produce the logos in Figure 9A and to generate the PSSM matrices used in Figure 9B–9D. A custom C code was used to scan the Suz12 sites with those PSSM matrices, assuming independent bases in the background. Support vector machine implemented in the e1071 R-package was used to classify the Suz12 binding sites with 10-fold cross validation.

### Accession number

The ChIP-seq data are available from Gene Expression Omnibus under access number GSE28325.

## Supporting Information

Figure S1 Suz12-TAP and Pcl3-V5 localize to the nucleus. (A) Schematic of recombination events performed to create *Suz12^Suz12TAP/+^*. The wild type endogenous allele is not pictured. Not to scale. (B) Staining of *Suz12^Suz12TAP/+^* with anti-FlagM2 (red), which overlays with nuclei stained with DAPI (blue). Scale bar 10 µm. (C) Suz12 expression levels in wild type, *Suz12^Gt/+^*, *Suz12^Rev/+^*, and *Suz12^Suz12TAP/+^* cell lines measured by qRT-PCR. Error bars indicate standard deviation. Asterisk denotes statistical significance of *p*<0.0001. Graph represents average expression of three different clones in three experiments assayed in quadruplet. (D) Wild type cells transfected with Pcl3-V5 and stained with anti-V5 (red). V5 staining overlays with nuclei stained with DAPI (blue). Scale bar 10 µm. Stainings were performed two times.
(TIF)

Figure S2 Depletion of Pcl3 does not affect ESC differentiation to all three germ layers. (A) Protein levels of Pcl3 in scramble and *Pcl3* shRNA clones assessed by immunoblot of lysates immunoprecipitated and probed with anti-Pcl3. For a positive control, Pcl3-TAP was immunoprecipitated and probed for FlagM2. Each lane represents a different clone. (B) Immunofluorescent staining of scramble and *Pcl3*-shRNA treated ESCs with Oct4, and Nanog. DAPI marks nuclei. Taken at 20×. (C) Scramble and *Pcl3* shRNA ESCs stained for alkaline phosphatase activity. View of one well of a 6-well plate and magnified at 16×. (D) *Pcl3* mRNA levels as measured by qRT-PCR in wild type and *Pcl3* overexpressing cells. (E) Detection of Pcl3-TAP in wild type cells expressing *Pcl3-TAP* by immunoprecipitating and probing with anti-FlagM2. (F) Images of colonies stained with methylene blue formed from scramble and *Pcl3* shRNA cells. Experiment was performed five times with two clones each of scramble and *Pcl3* shRNA ESCs. (G) *Pcl3* expression in scramble and *Pcl3*-shRNA derived teratomas. Representative of eight teratomas. (H) Teratomas expressing scramble and *Pcl3* shRNA stained for the neural marker NeuN (green) and the neuron marker Tuj1 (red); muscle marker Actin (green); basal layer skin marker K14 (green). All images contain nuclear staining with DAPI (blue). Scale bar 20 µm. (I) Expression levels of *Nestin*, *T-brachyury* (*T-bra*), *Hnf4*, and *Pcl3* which represent neuroectoderm, mesoderm, endoderm, and knockdown respectively, in scramble and *Pcl3* shRNA expressing EBs. Experiment was performed with 3–6 clones. All immunoblots were performed 2–4 times, and α-tubulin was used as a loading control. All stainings were performed 2–3 times in 2–4 clones or teratoma samples. Expression analysis represents 3–4 experiments assayed in quadruplicate. Error bars indicate standard deviation and asterisks indicate statistical significance of *p*<0.005.
(TIF)

Figure S3 Depletion of Suz12 and Pcl3. (A) Quantification of H3K27me3 depletion in *Pcl3* shRNA treated cells. Graph shows an approximate 80% decrease in H3K27me3 levels and represents ten experiments using 2–6 clones each. (B) qRT-PCR and (C) immunoblot indicating levels of Suz12 in cells treated with increasing amounts of *Suz12* siRNA 48 hrs and 72 hrs post-transfection. β-actin was used as a loading control. (D) Transfection with *Suz12* and *Pcl3* siRNAs causes decreased expression of *Suz12* and *Pcl3* respectively as measured by qRT-PCR. (E) Pcl3-TAP localizes to the nucleus as assessed by immunofluorescent overlay of FlagM2 (green) and DAPI (blue). E-cadherin (red) marks the cell membrane. Scale bar 10 µm. Expression analysis was performed 2–4 times and assayed in quadruplet. Error bars indicate standard deviation, and asterisks indicate statistical significance of *p*<0.005. Staining was performed twice with three different clones.
(TIF)

Figure S4 *Pcl3* knockdown causes the most significant depletion of Suz12 and H3K27me3 on chromosome 11. (A) Number of sequenced and aligned reads for ChIP-sequencing. (B) Partial sums of order statistics of binned read counts in the scramble and *Pcl3* shRNA cells were computed, and their ratios are plotted for the Suz12 FlagM2 ChIP and H3K27me3 ChIP. The theoretical ratio of partial sums is almost linear when two samples are identically distributed, as shown by the red line. The blue vertical bar marks the quantile at which the ratio begins to deviate from linearity, and it effectively separates the background bins from ChIP-enriched bins. The ratio at this quantile was used to scale the Pcl3 ChIP-seq counts. (C) Boxplot comparing Suz12 binding sites to Marson et al. [64]. The most significant Suz12 binding sites identified in our dataset overlap with Marson et al. while the less significant binding sites make up the majority of the remaining sites not identified in Marson et al. ChIP score = −log p-value for Suz12 ChIP-seq/Input. (D) Graph of −log p-values indicates that the most significant Suz12 ChIP-seq binding sites are more likely to decrease following *Pcl3* knockdown, while sites unaffected by *Pcl3* knockdown are most often less significant Suz12 ChIP-seq binding sites. (E) Boxplot of log fold-changes in ChIP-seq read density within Suz12 binding sites. Suz12 binding and H3K27me3 in *Pcl3* knockdown ESCs were decreased on all chromosomes. Chromosome 11 was the most significantly depleted (Pair-wise Wilcoxon rank sum test p-value<1.3×10^−15^ for Suz12 binding sites; p-value<5.2×10^−14^ for H3K27me3). (F) Sites with decreased Suz12 binding upon *Pcl3* knockdown tend to be devoid of E2f1 [63] (Fisher test p-value = 9.5×10^−53^). (G) Suz12 ChIP-qRT-PCR indicating that at some sites Pcl3 depletion does not affect Suz12 binding. (H) ChIP-qRT-PCR for H3K4me3 in scramble and *Pcl3* shRNA ESCs. Error bars indicate standard deviation, and asterisks indicate statistical significance of *p*<0.04. ChIP-qRT-PCRs was performed 2–3 times and assayed in quadruplicate.
(TIF)

Figure S5 Pcl3 co-localizes with Suz12. (A) Aligning Suz12 and Pcl3 ChIP-seq reads at Pcl3 peak centers shows genome-wide co-localization of Pcl3 with Suz12. (B) To estimate the percentage overlap between Suz12 and Pcl3 binding sites, a sensitivity analysis was performed by varying the Skellam distribution p-value cutoff for calling peaks, ranging between 10^−7^ to 10^−12^. The boxplot shows the percentage of Pcl3 peaks at each p-value cutoff found to be overlapping with Suz12 binding sites that pass the p-value cutoffs 10^−7^, 10^−8^, 10^−9^, 10^−10^, 10^−11^, and 10^−12^.
(TIF)

Figure S6 Pcl3 misregulates a subset of genes. (A, C) Relative expression of genes that either increased or decreased upon *Pcl3* knockdown as measured by qRT-PCR and microarray. Cells were collected following selection for Pcl3 depletion, approximately 3–4 weeks. Microarray and qRT-PCR were performed with 2–6 clones each for control and *Pcl3* knockdown ESCs. qRT-PCR analysis represents 2–3 different experiments performed with multiple clones and assayed in quadruplicate. For both graphs, error bars indicate standard deviation and data represents statistical significance of *p*<0.05. (A) Graph represents expression levels in ESCs that have not been pre-plated to remove differentiated cells. (B) Genes that show depleted Suz12 binding following *Pcl3* knockdown and that are misregulated by microarray analysis. Cells in (C) were pre-plated to remove differentiated cells and then expression was assessed. (D) Binding profile of Suz12 and Pcl3 at the *Suz12* locus. Turquoise puncta are background and not statistically significant.
(TIF)

Table S1 Gene expression changes upon *Pcl3* knockdown by microarray. Microarray data indicating fold changes in gene expression in ESCs expressing *Pcl3* shRNA as compared to scramble shRNA ESCs. Comparison included six control samples and six *Pcl3* shRNA clones.
(XLS)

Table S2 Gene expression changes in pre-plated *Pcl3* shRNA ESCs by microarray. Microarray data indicating fold changes in gene expression in pre-plated ESCs expressing *Pcl3* shRNA as compared to scramble shRNA ESCs. Comparison included two scramble clones and two *Pcl3* shRNA clones.
(XLS)

Text S1 Supplemental methods. Detailed description of the microarray and ChIP-sequencing methods as well as a list of the antibodies and primers used.
(DOC)

## References

[1] Surface LE, Thornton SR, Boyer LA (2010). Polycomb group proteins set the stage for early lineage commitment.. Cell Stem Cell.

[2] Margueron R, Reinberg D (2011). The Polycomb complex PRC2 and its mark in life.. Nature.

[3] Kuzmichev A, Nishioka K, Erdjument-Bromage H, Tempst P, Reinberg D (2002). Histone methyltransferase activity associated with a human multiprotein complex containing the Enhancer of Zeste protein.. Genes Dev.

[4] Cao R, Wang L, Wang H, Xia L, Erdjument-Bromage H (2002). Role of histone H3 lysine 27 methylation in Polycomb-group silencing.. Science.

[5] Czermin B, Melfi R, McCabe D, Seitz V, Imhof A (2002). Drosophila enhancer of Zeste/ESC complexes have a histone H3 methyltransferase activity that marks chromosomal Polycomb sites.. Cell.

[6] Muller J, Hart CM, Francis NJ, Vargas ML, Sengupta A (2002). Histone methyltransferase activity of a Drosophila Polycomb group repressor complex.. Cell.

[7] Kirmizis A, Bartley SM, Kuzmichev A, Margueron R, Reinberg D (2004). Silencing of human polycomb target genes is associated with methylation of histone H3 Lys 27.. Genes Dev.

[8] Mozzetta C, Consalvi S, Saccone V, Forcales SV, Puri PL (2011). Selective control of Pax7 expression by TNF-activated p38alpha/polycomb repressive complex 2 (PRC2) signaling during muscle satellite cell differentiation.. Cell Cycle.

[9] Khromov T, Pantakani DV, Nolte J, Wolf M, Dressel R (2011). Global and gene-specific histone modification profiles of mouse multipotent adult germline stem cells.. Mol Hum Reprod.

[10] Oguro H, Yuan J, Ichikawa H, Ikawa T, Yamazaki S (2010). Poised lineage specification in multipotential hematopoietic stem and progenitor cells by the polycomb protein Bmi1.. Cell Stem Cell.

[11] Bernstein BE, Mikkelsen TS, Xie X, Kamal M, Huebert DJ (2006). A bivalent chromatin structure marks key developmental genes in embryonic stem cells.. Cell.

[12] Jorgensen HF, Giadrossi S, Casanova M, Endoh M, Koseki H (2006). Stem cells primed for action: polycomb repressive complexes restrain the expression of lineage-specific regulators in embryonic stem cells.. Cell Cycle.

[13] Ku M, Koche RP, Rheinbay E, Mendenhall EM, Endoh M (2008). Genomewide analysis of PRC1 and PRC2 occupancy identifies two classes of bivalent domains.. PLoS Genet.

[14] Azuara V, Perry P, Sauer S, Spivakov M, Jorgensen HF (2006). Chromatin signatures of pluripotent cell lines.. Nat Cell Biol.

[15] Lim DA, Huang YC, Swigut T, Mirick AL, Garcia-Verdugo JM (2009). Chromatin remodelling factor Mll1 is essential for neurogenesis from postnatal neural stem cells.. Nature.

[16] Zhu J, He F, Hu S, Yu J (2008). On the nature of human housekeeping genes.. Trends Genet.

[17] Saxonov S, Berg P, Brutlag DL (2006). A genome-wide analysis of CpG dinucleotides in the human genome distinguishes two distinct classes of promoters.. Proc Natl Acad Sci U S A.

[18] Larsen F, Gundersen G, Lopez R, Prydz H (1992). CpG islands as gene markers in the human genome.. Genomics.

[19] Mendenhall EM, Koche RP, Truong T, Zhou VW, Issac B (2010). GC-rich sequence elements recruit PRC2 in mammalian ES cells.. PLoS Genet.

[20] Thomson JP, Skene PJ, Selfridge J, Clouaire T, Guy J (2010). CpG islands influence chromatin structure via the CpG-binding protein Cfp1.. Nature.

[21] Leeb M, Pasini D, Novatchkova M, Jaritz M, Helin K (2010). Polycomb complexes act redundantly to repress genomic repeats and genes.. Genes Dev.

[22] Chamberlain SJ, Yee D, Magnuson T (2008). Polycomb repressive complex 2 is dispensable for maintenance of embryonic stem cell pluripotency.. Stem Cells.

[23] Pasini D, Bracken AP, Jensen MR, Lazzerini Denchi E, Helin K (2004). Suz12 is essential for mouse development and for EZH2 histone methyltransferase activity.. Embo J.

[24] Lee TI, Jenner RG, Boyer LA, Guenther MG, Levine SS (2006). Control of developmental regulators by Polycomb in human embryonic stem cells.. Cell.

[25] Montgomery ND, Yee D, Chen A, Kalantry S, Chamberlain SJ (2005). The murine polycomb group protein Eed is required for global histone H3 lysine-27 methylation.. Curr Biol.

[26] Faust C, Schumacher A, Holdener B, Magnuson T (1995). The eed mutation disrupts anterior mesoderm production in mice.. Development.

[27] O'Carroll D, Erhardt S, Pagani M, Barton SC, Surani MA (2001). The polycomb-group gene Ezh2 is required for early mouse development.. Mol Cell Biol.

[28] Cao R, Zhang Y (2004). SUZ12 is required for both the histone methyltransferase activity and the silencing function of the EED-EZH2 complex.. Mol Cell.

[29] Pasini D, Bracken AP, Hansen JB, Capillo M, Helin K (2007). The polycomb group protein Suz12 is required for embryonic stem cell differentiation.. Mol Cell Biol.

[30] Shen X, Liu Y, Hsu YJ, Fujiwara Y, Kim J (2008). EZH1 mediates methylation on histone H3 lysine 27 and complements EZH2 in maintaining stem cell identity and executing pluripotency.. Mol Cell.

[31] Sauvageau M, Sauvageau G (2010). Polycomb group proteins: multi-faceted regulators of somatic stem cells and cancer.. Cell Stem Cell.

[32] Valk-Lingbeek ME, Bruggeman SW, van Lohuizen M (2004). Stem cells and cancer; the polycomb connection.. Cell.

[33] Sparmann A, van Lohuizen M (2006). Polycomb silencers control cell fate, development and cancer.. Nat Rev Cancer.

[34] Wang S, Robertson GP, Zhu J (2004). A novel human homologue of Drosophila polycomblike gene is up-regulated in multiple cancers.. Gene.

[35] Kim H, Kang K, Kim J (2009). AEBP2 as a potential targeting protein for Polycomb Repression Complex PRC2.. Nucleic Acids Res.

[36] Peng JC, Valouev A, Swigut T, Zhang J, Zhao Y (2009). Jarid2/Jumonji coordinates control of PRC2 enzymatic activity and target gene occupancy in pluripotent cells.. Cell.

[37] Shen X, Kim W, Fujiwara Y, Simon MD, Liu Y (2009). Jumonji modulates polycomb activity and self-renewal versus differentiation of stem cells.. Cell.

[38] Pasini D, Cloos PA, Walfridsson J, Olsson L, Bukowski JP (2010). JARID2 regulates binding of the Polycomb repressive complex 2 to target genes in ES cells.. Nature.

[39] Li G, Margueron R, Ku M, Chambon P, Bernstein BE (2010). Jarid2 and PRC2, partners in regulating gene expression.. Genes Dev.

[40] Landeira D, Sauer S, Poot R, Dvorkina M, Mazzarella L (2010). Jarid2 is a PRC2 component in embryonic stem cells required for multi-lineage differentiation and recruitment of PRC1 and RNA Polymerase II to developmental regulators.. Nat Cell Biol.

[41] Walker E, Chang WY, Hunkapiller J, Cagney G, Garcha K (2010). Polycomb-like 2 associates with PRC2 and regulates transcriptional networks during mouse embryonic stem cell self-renewal and differentiation.. Cell Stem Cell.

[42] Tie F, Prasad-Sinha J, Birve A, Rasmuson-Lestander A, Harte PJ (2003). A 1-megadalton ESC/E(Z) complex from Drosophila that contains polycomblike and RPD3.. Mol Cell Biol.

[43] Lonie A, D'Andrea R, Paro R, Saint R (1994). Molecular characterisation of the Polycomblike gene of Drosophila melanogaster, a trans-acting negative regulator of homeotic gene expression.. Development.

[44] Duncan IM (1982). Polycomblike: a gene that appears to be required for the normal expression of the bithorax and antennapedia gene complexes of Drosophila melanogaster.. Genetics.

[45] O'Connell S, Wang L, Robert S, Jones CA, Saint R (2001). Polycomblike PHD fingers mediate conserved interaction with enhancer of zeste protein.. J Biol Chem.

[46] Savla U, Benes J, Zhang J, Jones RS (2008). Recruitment of Drosophila Polycomb-group proteins by Polycomblike, a component of a novel protein complex in larvae.. Development.

[47] Li X, Isono K, Yamada D, Endo TA, Endoh M (2011). Mammalian Polycomb-Like Pcl2/Mtf2 Is a Novel Regulatory Component of PRC2 That Can Differentially Modulate Polycomb Activity both at the Hox Gene Cluster and at Cdkn2a Genes.. Mol Cell Biol.

[48] Zhang Z, Jones A, Sun CW, Li C, Chang CW (2011). PRC2 Complexes with JARID2, MTF2, and esPRC2p48 in ES Cells to Modulate ES Cell Pluripotency and Somatic Cell Reprograming.. Stem Cells.

[49] Coulson M, Robert S, Eyre HJ, Saint R (1998). The identification and localization of a human gene with sequence similarity to Polycomblike of Drosophila melanogaster.. Genomics.

[50] Nekrasov M, Klymenko T, Fraterman S, Papp B, Oktaba K (2007). Pcl-PRC2 is needed to generate high levels of H3-K27 trimethylation at Polycomb target genes.. Embo J.

[51] Sarma K, Margueron R, Ivanov A, Pirrotta V, Reinberg D (2008). Ezh2 requires PHF1 to efficiently catalyze H3 lysine 27 trimethylation in vivo.. Mol Cell Biol.

[52] Cao R, Wang H, He J, Erdjument-Bromage H, Tempst P (2008). Role of hPHF1 in H3K27 methylation and Hox gene silencing.. Mol Cell Biol.

[53] Boulay G, Rosnoblet C, Guerardel C, Angrand PO, Leprince D (2011). Functional characterization of hPCL3 (human Polycomb-like 3) isoforms identifies them as components of distinct EZH2 protein complexes.. Biochem J.

[54] Singla V, Hunkapiller J, Santos N, Seol AD, Norman AR (2010). Floxin, a resource for genetically engineering mouse ESCs.. Nat Methods.

[55] Boyer LA, Plath K, Zeitlinger J, Brambrink T, Medeiros LA (2006). Polycomb complexes repress developmental regulators in murine embryonic stem cells.. Nature.

[56] Fazzio TG, Huff JT, Panning B (2008). An RNAi screen of chromatin proteins identifies Tip60-p400 as a regulator of embryonic stem cell identity.. Cell.

[57] Foley E, O'Farrell PH (2004). Functional dissection of an innate immune response by a genome-wide RNAi screen.. PLoS Biol.

[58] de Napoles M, Mermoud JE, Wakao R, Tang YA, Endoh M (2004). Polycomb group proteins Ring1A/B link ubiquitylation of histone H2A to heritable gene silencing and X inactivation.. Dev Cell.

[59] de la Cruz CC, Kirmizis A, Simon MD, Isono K, Koseki H (2007). The polycomb group protein SUZ12 regulates histone H3 lysine 9 methylation and HP1 alpha distribution.. Chromosome Res.

[60] Rea S, Eisenhaber F, O'Carroll D, Strahl BD, Sun ZW (2000). Regulation of chromatin structure by site-specific histone H3 methyltransferases.. Nature.

[61] Pasini D, Malatesta M, Jung HR, Walfridsson J, Willer A (2010). Characterization of an antagonistic switch between histone H3 lysine 27 methylation and acetylation in the transcriptional regulation of Polycomb group target genes.. Nucleic Acids Res.

[62] Langmead B, Trapnell C, Pop M, Salzberg SL (2009). Ultrafast and memory-efficient alignment of short DNA sequences to the human genome.. Genome Biol.

[63] Chen X, Xu H, Yuan P, Fang F, Huss M (2008). Integration of external signaling pathways with the core transcriptional network in embryonic stem cells.. Cell.

[64] Marson A, Levine SS, Cole MF, Frampton GM, Brambrink T (2008). Connecting microRNA genes to the core transcriptional regulatory circuitry of embryonic stem cells.. Cell.

[65] Mikkelsen TS, Ku M, Jaffe DB, Issac B, Lieberman E (2007). Genome-wide maps of chromatin state in pluripotent and lineage-committed cells.. Nature.

[66] Tzur G, Levy A, Meiri E, Barad O, Spector Y (2008). MicroRNA expression patterns and function in endodermal differentiation of human embryonic stem cells.. PLoS ONE.

[67] Barber BA, Rastegar M (2010). Epigenetic control of Hox genes during neurogenesis, development, and disease.. Ann Anat.

[68] Huang H, Xie C, Sun X, Ritchie RP, Zhang J (2010). miR-10a contributes to retinoid acid-induced smooth muscle cell differentiation.. J Biol Chem.

[69] Tarantino C, Paolella G, Cozzuto L, Minopoli G, Pastore L (2010). miRNA 34a, 100, and 137 modulate differentiation of mouse embryonic stem cells.. Faseb J.

[70] Kim YJ, Bae SW, Yu SS, Bae YC, Jung JS (2009). miR-196a regulates proliferation and osteogenic differentiation in mesenchymal stem cells derived from human adipose tissue.. J Bone Miner Res.

[71] Brown JL, Snir M, Noushmehr H, Kirby M, Hong SK (2008). Transcriptional profiling of endogenous germ layer precursor cells identifies dusp4 as an essential gene in zebrafish endoderm specification.. Proc Natl Acad Sci U S A.

[72] Mallo M, Wellik DM, Deschamps J (2010). Hox genes and regional patterning of the vertebrate body plan.. Dev Biol.

[73] Ucar A, Vafaizadeh V, Jarry H, Fiedler J, Klemmt PA (2010). miR-212 and miR-132 are required for epithelial stromal interactions necessary for mouse mammary gland development.. Nat Genet.

[74] Sato F, Tsuchiya S, Meltzer SJ, Shimizu K (2011). MicroRNAs and epigenetics.. Febs J.

[75] Pizzatti L, Binato R, Cofre J, Gomes BE, Dobbin J (2010). SUZ12 is a candidate target of the non-canonical WNT pathway in the progression of chronic myeloid leukemia.. Genes Chromosomes Cancer.

[76] Kirmizis A, Bartley SM, Farnham PJ (2003). Identification of the polycomb group protein SU(Z)12 as a potential molecular target for human cancer therapy.. Mol Cancer Ther.

[77] Casanova M, Preissner T, Cerase A, Poot R, Yamada D (2011). Polycomblike 2 facilitates the recruitment of PRC2 Polycomb group complexes to the inactive X chromosome and to target loci in embryonic stem cells.. Development.

[78] Friberg A, Oddone A, Klymenko T, Muller J, Sattler M (2010). Structure of an atypical Tudor domain in the Drosophila Polycomblike protein.. Protein Sci.

[79] Walker E, Manias JL, Chang WY, Stanford WL (2011). PCL2 modulates gene regulatory networks controlling self-renewal and commitment in embryonic stem cells.. Cell Cycle.

[80] Sing A, Pannell D, Karaiskakis A, Sturgeon K, Djabali M (2009). A vertebrate Polycomb response element governs segmentation of the posterior hindbrain.. Cell.

[81] Chan CS, Rastelli L, Pirrotta V (1994). A Polycomb response element in the Ubx gene that determines an epigenetically inherited state of repression.. Embo J.

[82] Muller J, Kassis JA (2006). Polycomb response elements and targeting of Polycomb group proteins in Drosophila.. Curr Opin Genet Dev.

[83] Kerppola TK (2009). Polycomb group complexes–many combinations, many functions.. Trends Cell Biol.

[84] Woo CJ, Kharchenko PV, Daheron L, Park PJ, Kingston RE (2010). A region of the human HOXD cluster that confers polycomb-group responsiveness.. Cell.

[85] Tanay A, O'Donnell AH, Damelin M, Bestor TH (2007). Hyperconserved CpG domains underlie Polycomb-binding sites.. Proc Natl Acad Sci U S A.

[86] Carroll JS, Meyer CA, Song J, Li W, Geistlinger TR (2006). Genome-wide analysis of estrogen receptor binding sites.. Nat Genet.

[87] Zhao J, Sun BK, Erwin JA, Song JJ, Lee JT (2008). Polycomb proteins targeted by a short repeat RNA to the mouse X chromosome.. Science.

[88] Gupta RA, Shah N, Wang KC, Kim J, Horlings HM (2010). Long non-coding RNA HOTAIR reprograms chromatin state to promote cancer metastasis.. Nature.

[89] Tsai MC, Manor O, Wan Y, Mosammaparast N, Wang JK (2010). Long noncoding RNA as modular scaffold of histone modification complexes.. Science.

[90] Kim J, Daniel J, Espejo A, Lake A, Krishna M (2006). Tudor, MBT and chromo domains gauge the degree of lysine methylation.. EMBO Rep.

[91] Sprangers R, Groves MR, Sinning I, Sattler M (2003). High-resolution X-ray and NMR structures of the SMN Tudor domain: conformational variation in the binding site for symmetrically dimethylated arginine residues.. J Mol Biol.

[92] Lan F, Collins RE, De Cegli R, Alpatov R, Horton JR (2007). Recognition of unmethylated histone H3 lysine 4 links BHC80 to LSD1-mediated gene repression.. Nature.

[93] Guenther MG, Levine SS, Boyer LA, Jaenisch R, Young RA (2007). A chromatin landmark and transcription initiation at most promoters in human cells.. Cell.

[94] Ohm JE, McGarvey KM, Yu X, Cheng L, Schuebel KE (2007). A stem cell-like chromatin pattern may predispose tumor suppressor genes to DNA hypermethylation and heritable silencing.. Nat Genet.

[95] Widschwendter M, Fiegl H, Egle D, Mueller-Holzner E, Spizzo G (2007). Epigenetic stem cell signature in cancer.. Nat Genet.

[96] Chase A, Cross NC (2011). Aberrations of EZH2 in cancer.. Clin Cancer Res.

[97] Singla V, Romaguera-Ros M, Garcia-Verdugo JM, Reiter JF (2010). Ofd1, a human disease gene, regulates the length and distal structure of centrioles.. Dev Cell.

[98] Shechter D, Dormann HL, Allis CD, Hake SB (2007). Extraction, purification and analysis of histones.. Nat Protoc.

[99] Hunkapiller J, Singla V, Seol A, Reiter JF (2010). The Ciliogenic Protein Ofd1 Regulates the Neuronal Differentiation of Embryonic Stem Cells.. Stem Cells Dev.

[100] Hawkins RD, Hon GC, Lee LK, Ngo Q, Lister R (2010). Distinct epigenomic landscapes of pluripotent and lineage-committed human cells.. Cell Stem Cell.

