# Supplementary figures and images for: Polycomb-Like 3 Promotes Polycomb Repressive Complex 2 Binding to CpG Islands and Embryonic Stem Cell Self-Renewal
Source: PLoS Genet. 2012 Mar 15;8(3):e1002576. doi: 10.1371/journal.pgen.1002576 (PMC3305387; doi:10.1371/journal.pgen.1002576)

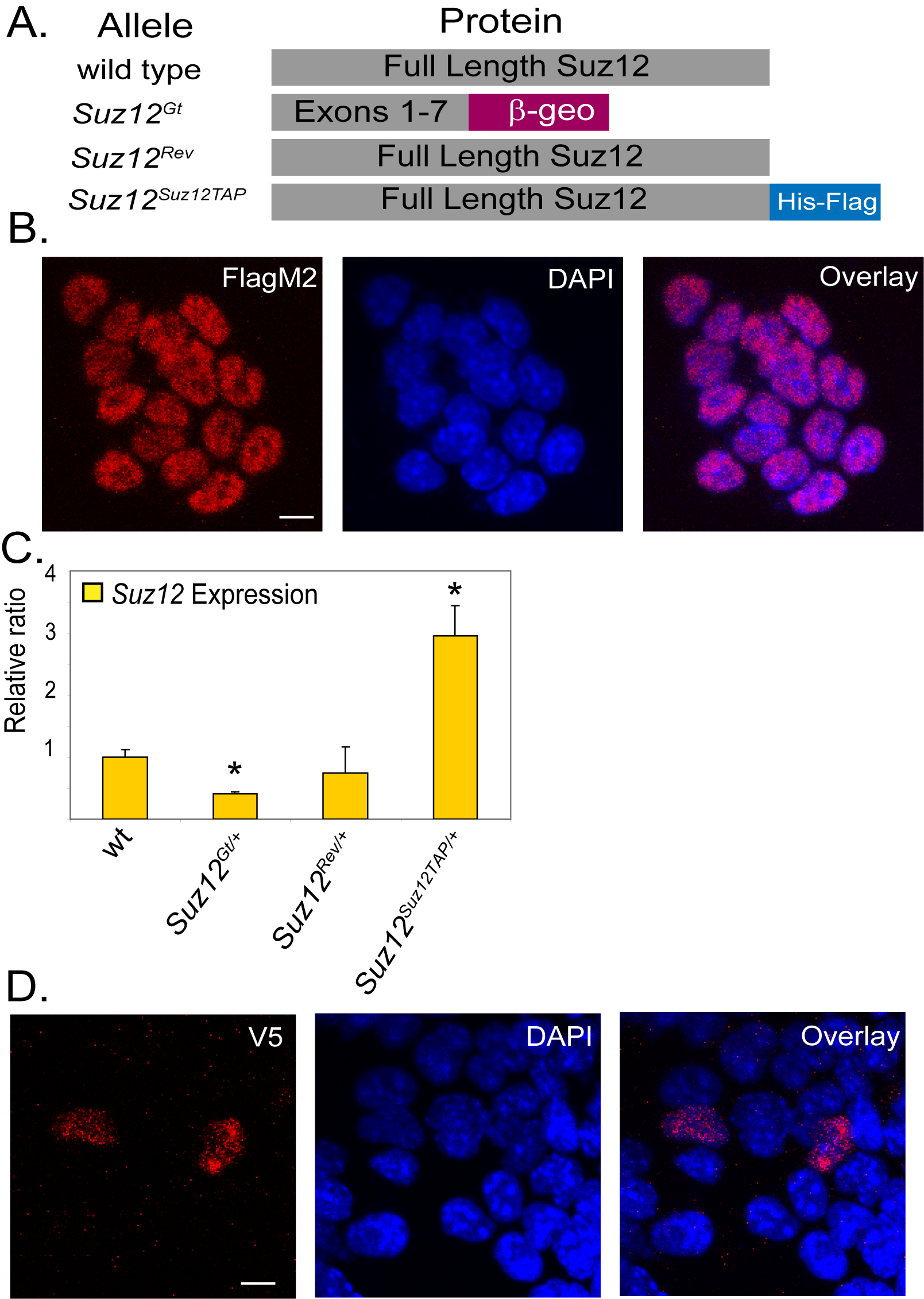

Supplement: Figure S1 — Suz12-TAP and Pcl3-V5 localize to the nucleus. (A) Schematic of recombination events performed to create Suz12Suz12TAP/+. The wild type endogenous allele is not pictured. Not to scale. (B) Staining of Suz12Suz12TAP/+ with anti-FlagM2 (red), which overlays with nuclei stained with DAPI (blue). Scale bar 10 µm. (C) Suz12 expression levels in wild type, Suz12Gt/+, Suz12Rev/+, and Suz12Suz12TAP/+ cell lines measured by qRT-PCR. Error bars indicate standard deviation. Asterisk denotes statistical significance of p<0.0001. Graph represents average expression of three different clones in three experiments assayed in quadruplet. (D) Wild type cells transfected with Pcl3-V5 and stained with anti-V5 (red). V5 staining overlays with nuclei stained with DAPI (blue). Scale bar 10 µm. Stainings were performed two times. (TIF) [file pgen.1002576.s001.tif]

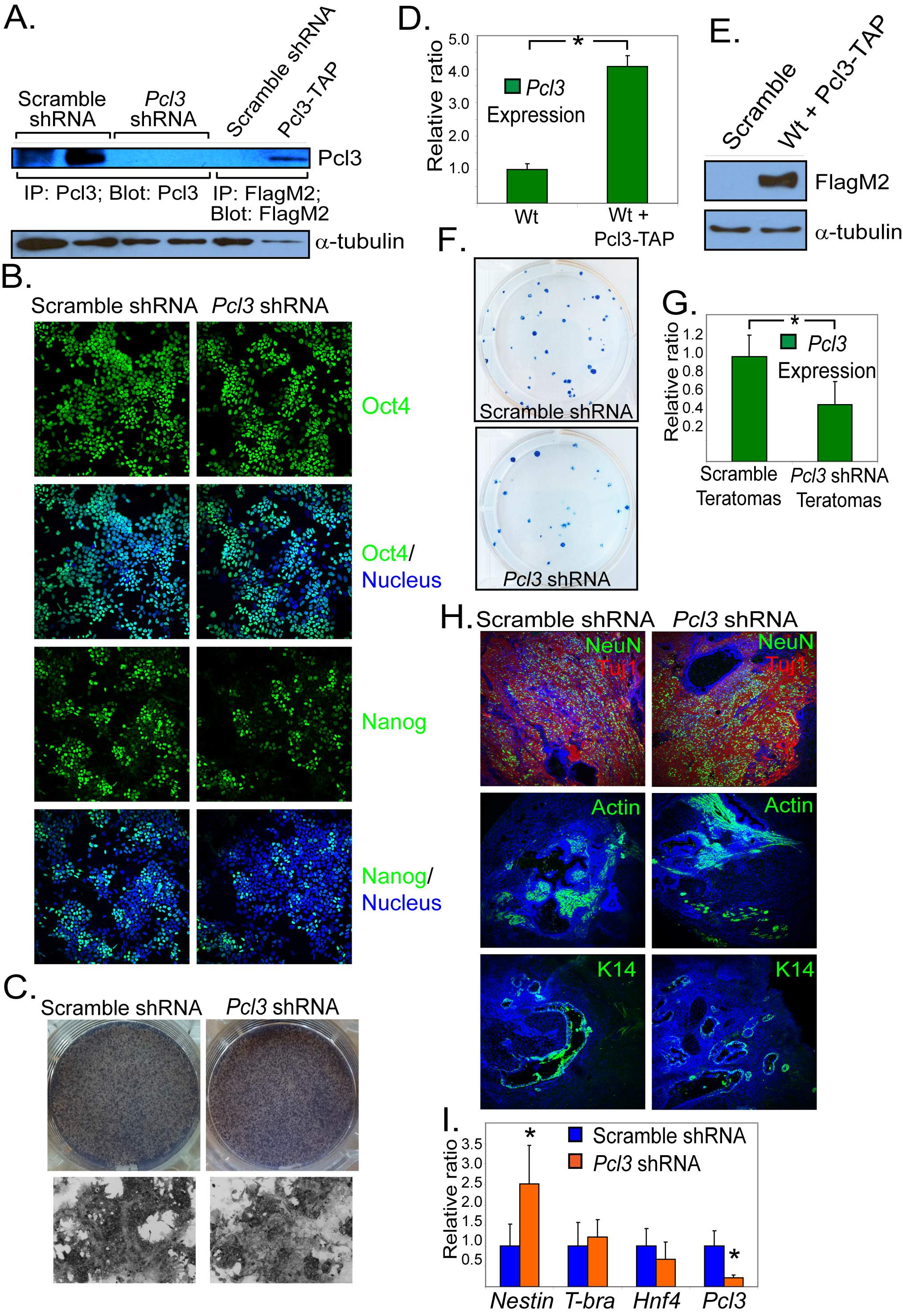

Supplement: Figure S2 — Depletion of Pcl3 does not affect ESC differentiation to all three germ layers. (A) Protein levels of Pcl3 in scramble and Pcl3 shRNA clones assessed by immunoblot of lysates immunoprecipitated and probed with anti-Pcl3. For a positive control, Pcl3-TAP was immunoprecipitated and probed for FlagM2. Each lane represents a different clone. (B) Immunofluorescent staining of scramble and Pcl3-shRNA treated ESCs with Oct4, and Nanog. DAPI marks nuclei. Taken at 20×. (C) Scramble and Pcl3 shRNA ESCs stained for alkaline phosphatase activity. View of one well of a 6-well plate and magnified at 16×. (D) Pcl3 mRNA levels as measured by qRT-PCR in wild type and Pcl3 overexpressing cells. (E) Detection of Pcl3-TAP in wild type cells expressing Pcl3-TAP by immunoprecipitating and probing with anti-FlagM2. (F) Images of colonies stained with methylene blue formed from scramble and Pcl3 shRNA cells. Experiment was performed five times with two clones each of scramble and Pcl3 shRNA ESCs. (G) Pcl3 expression in scramble and Pcl3-shRNA derived teratomas. Representative of eight teratomas. (H) Teratomas expressing scramble and Pcl3 shRNA stained for the neural marker NeuN (green) and the neuron marker Tuj1 (red); muscle marker Actin (green); basal layer skin marker K14 (green). All images contain nuclear staining with DAPI (blue). Scale bar 20 µm. (I) Expression levels of Nestin, T-brachyury (T-bra), Hnf4, and Pcl3 which represent neuroectoderm, mesoderm, endoderm, and knockdown respectively, in scramble and Pcl3 shRNA expressing EBs. Experiment was performed with 3–6 clones. All immunoblots were performed 2–4 times, and α-tubulin was used as a loading control. All stainings were performed 2–3 times in 2–4 clones or teratoma samples. Expression analysis represents 3–4 experiments assayed in quadruplicate. Error bars indicate standard deviation and asterisks indicate statistical significance of p<0.005. (TIF) [file pgen.1002576.s002.tif]

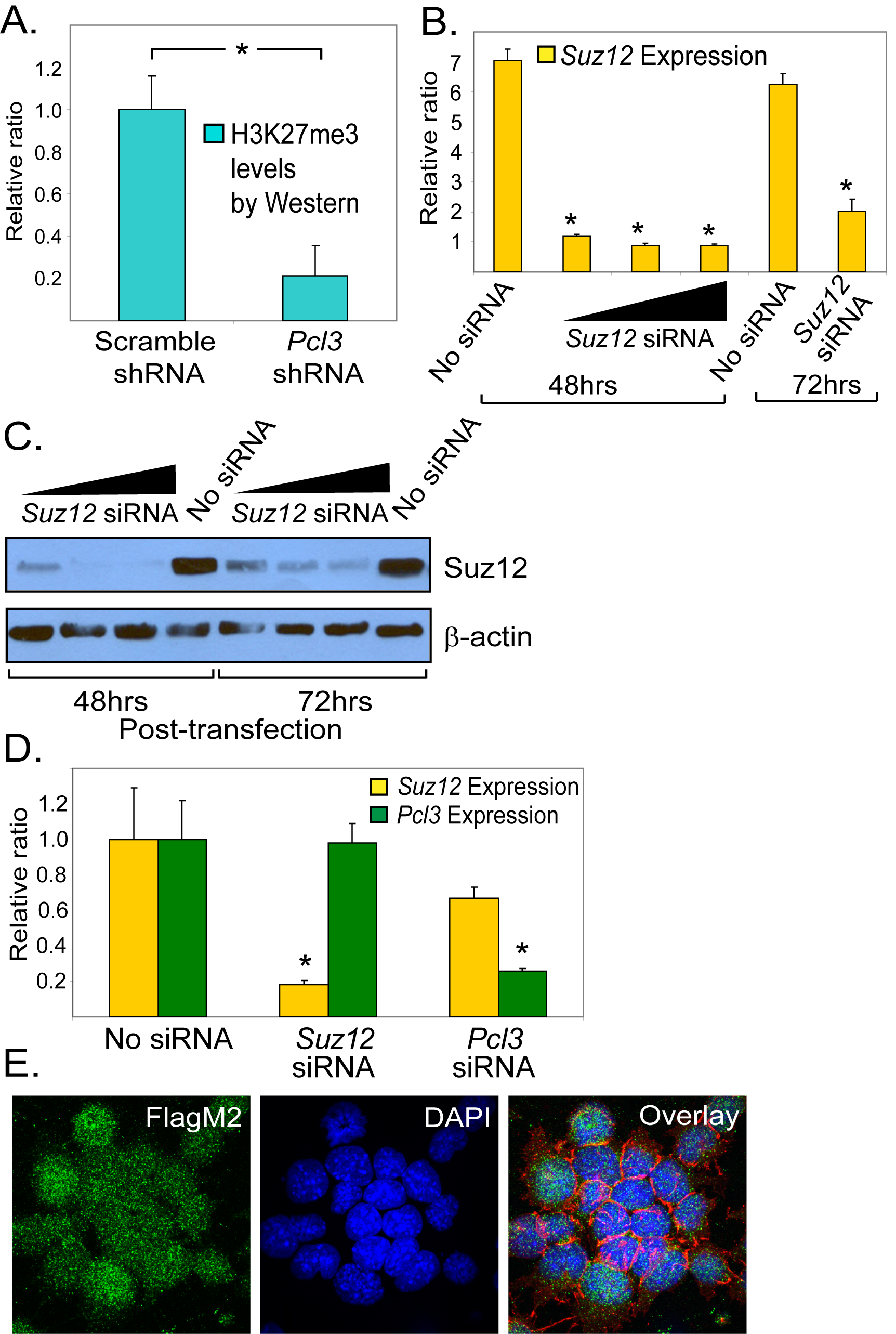

Supplement: Figure S3 — Depletion of Suz12 and Pcl3. (A) Quantification of H3K27me3 depletion in Pcl3 shRNA treated cells. Graph shows an approximate 80% decrease in H3K27me3 levels and represents ten experiments using 2–6 clones each. (B) qRT-PCR and (C) immunoblot indicating levels of Suz12 in cells treated with increasing amounts of Suz12 siRNA 48 hrs and 72 hrs post-transfection. β-actin was used as a loading control. (D) Transfection with Suz12 and Pcl3 siRNAs causes decreased expression of Suz12 and Pcl3 respectively as measured by qRT-PCR. (E) Pcl3-TAP localizes to the nucleus as assessed by immunofluorescent overlay of FlagM2 (green) and DAPI (blue). E-cadherin (red) marks the cell membrane. Scale bar 10 µm. Expression analysis was performed 2–4 times and assayed in quadruplet. Error bars indicate standard deviation, and asterisks indicate statistical significance of p<0.005. Staining was performed twice with three different clones. (TIF) [file pgen.1002576.s003.tif]

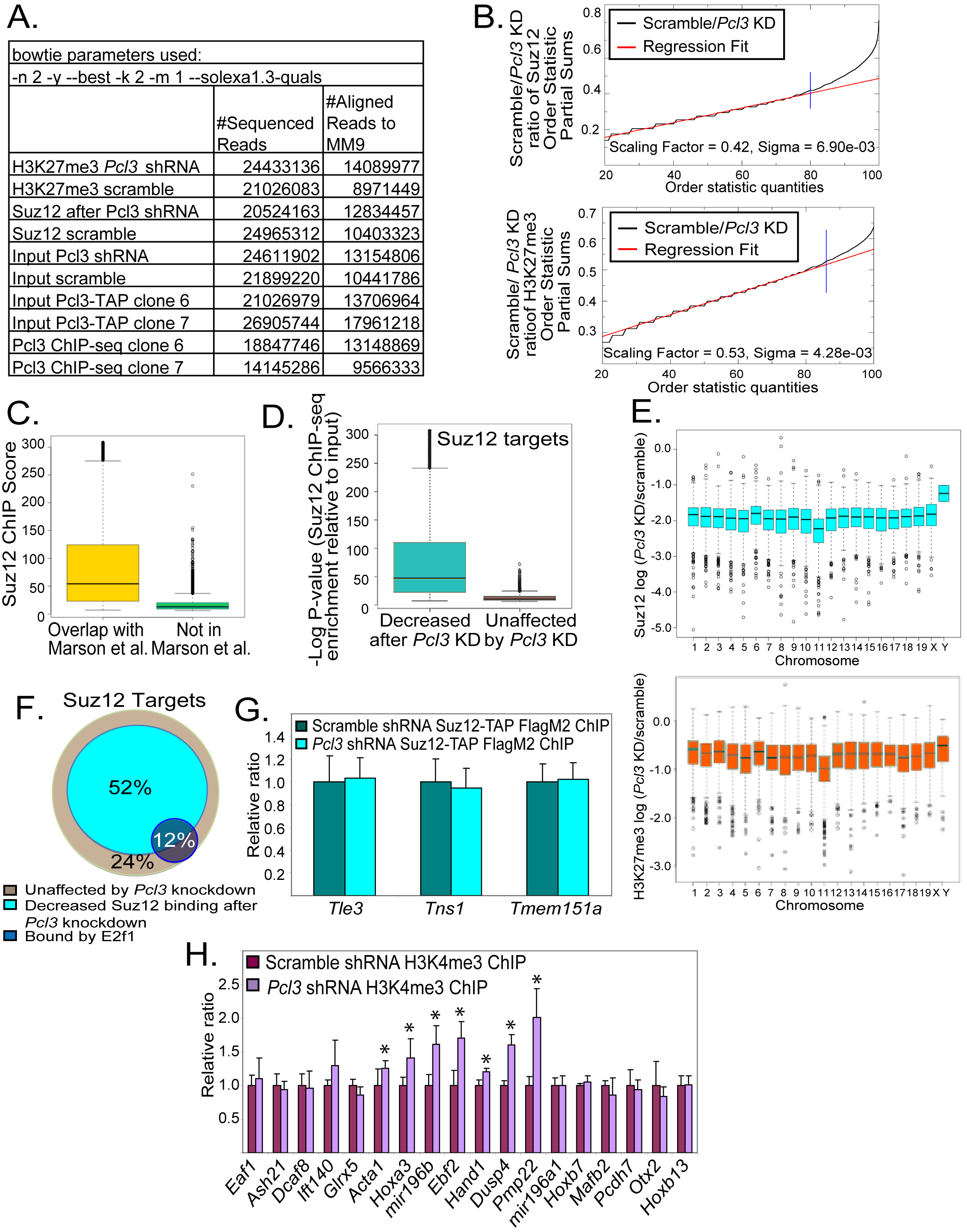

Supplement: Figure S4 — Pcl3 knockdown causes the most significant depletion of Suz12 and H3K27me3 on chromosome 11. (A) Number of sequenced and aligned reads for ChIP-sequencing. (B) Partial sums of order statistics of binned read counts in the scramble and Pcl3 shRNA cells were computed, and their ratios are plotted for the Suz12 FlagM2 ChIP and H3K27me3 ChIP. The theoretical ratio of partial sums is almost linear when two samples are identically distributed, as shown by the red line. The blue vertical bar marks the quantile at which the ratio begins to deviate from linearity, and it effectively separates the background bins from ChIP-enriched bins. The ratio at this quantile was used to scale the Pcl3 ChIP-seq counts. (C) Boxplot comparing Suz12 binding sites to Marson et al. [64]. The most significant Suz12 binding sites identified in our dataset overlap with Marson et al. while the less significant binding sites make up the majority of the remaining sites not identified in Marson et al. ChIP score = −log p-value for Suz12 ChIP-seq/Input. (D) Graph of −log p-values indicates that the most significant Suz12 ChIP-seq binding sites are more likely to decrease following Pcl3 knockdown, while sites unaffected by Pcl3 knockdown are most often less significant Suz12 ChIP-seq binding sites. (E) Boxplot of log fold-changes in ChIP-seq read density within Suz12 binding sites. Suz12 binding and H3K27me3 in Pcl3 knockdown ESCs were decreased on all chromosomes. Chromosome 11 was the most significantly depleted (Pair-wise Wilcoxon rank sum test p-value<1.3×10^−15^ for Suz12 binding sites; p-value<5.2×10^−14^ for H3K27me3). (F) Sites with decreased Suz12 binding upon Pcl3 knockdown tend to be devoid of E2f1 [63] (Fisher test p-value = 9.5×10^−53^). (G) Suz12 ChIP-qRT-PCR indicating that at some sites Pcl3 depletion does not affect Suz12 binding. (H) ChIP-qRT-PCR for H3K4me3 in scramble and Pcl3 shRNA ESCs. Error bars indicate standard deviation, and asterisks indicate statistical significance of p<0.04. C [file pgen.1002576.s004.tif]

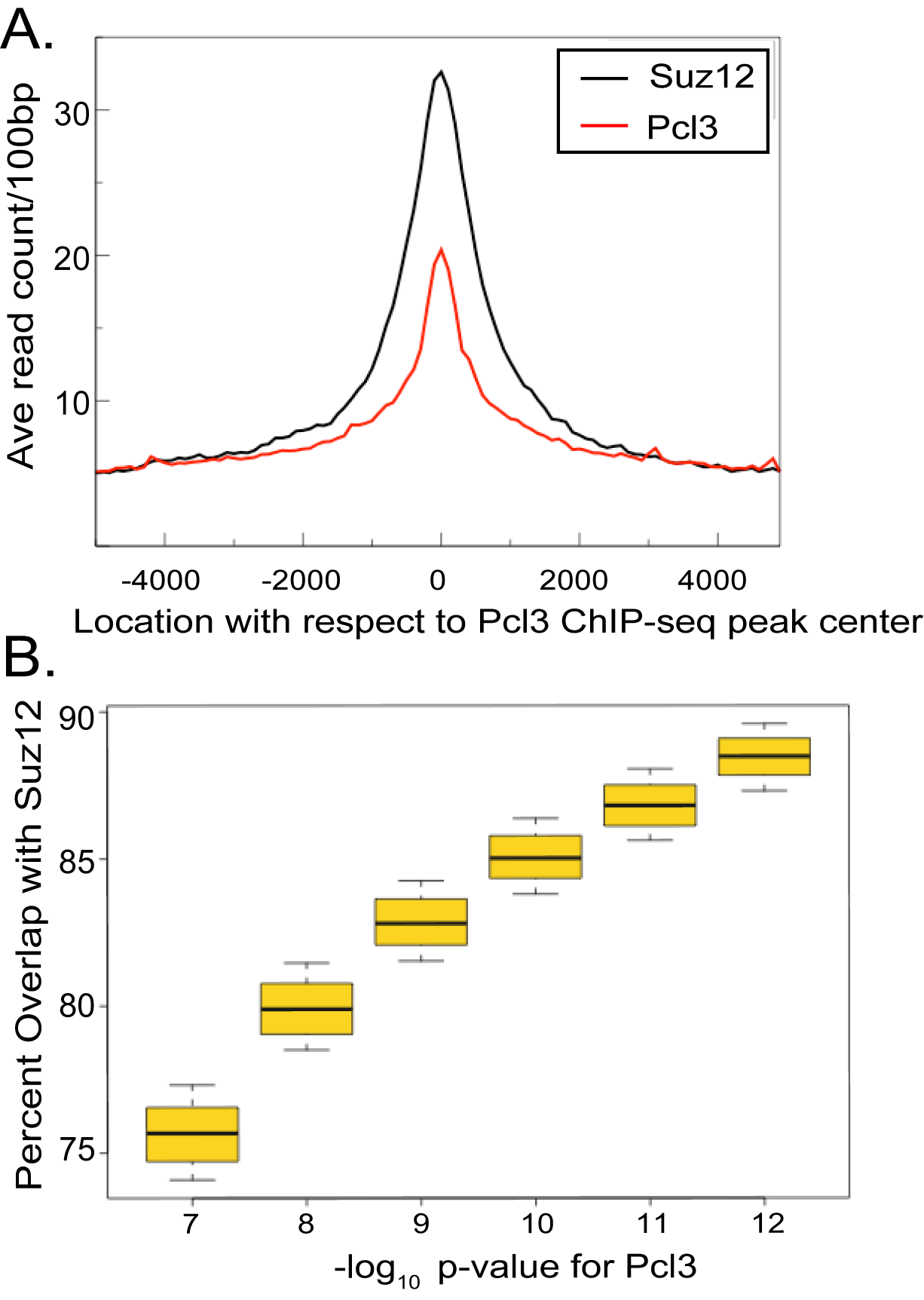

Supplement: Figure S5 — Pcl3 co-localizes with Suz12. (A) Aligning Suz12 and Pcl3 ChIP-seq reads at Pcl3 peak centers shows genome-wide co-localization of Pcl3 with Suz12. (B) To estimate the percentage overlap between Suz12 and Pcl3 binding sites, a sensitivity analysis was performed by varying the Skellam distribution p-value cutoff for calling peaks, ranging between 10−7 to 10−12. The boxplot shows the percentage of Pcl3 peaks at each p-value cutoff found to be overlapping with Suz12 binding sites that pass the p-value cutoffs 10−7, 10−8, 10−9, 10−10, 10−11, and 10−12. (TIF) [file pgen.1002576.s005.tif]

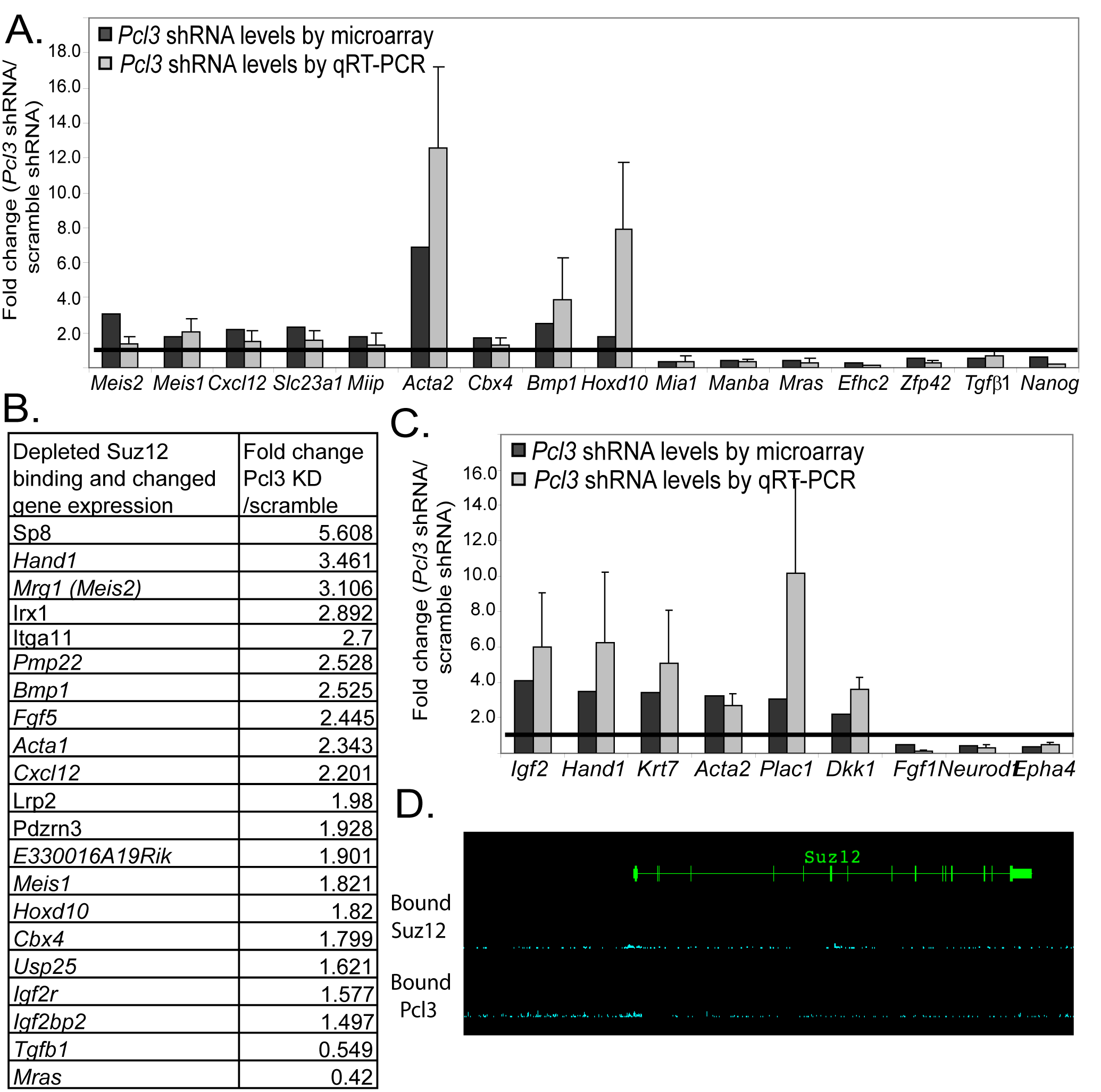

Supplement: Figure S6 — Pcl3 misregulates a subset of genes. (A, C) Relative expression of genes that either increased or decreased upon Pcl3 knockdown as measured by qRT-PCR and microarray. Cells were collected following selection for Pcl3 depletion, approximately 3–4 weeks. Microarray and qRT-PCR were performed with 2–6 clones each for control and Pcl3 knockdown ESCs. qRT-PCR analysis represents 2–3 different experiments performed with multiple clones and assayed in quadruplicate. For both graphs, error bars indicate standard deviation and data represents statistical significance of p<0.05. (A) Graph represents expression levels in ESCs that have not been pre-plated to remove differentiated cells. (B) Genes that show depleted Suz12 binding following Pcl3 knockdown and that are misregulated by microarray analysis. Cells in (C) were pre-plated to remove differentiated cells and then expression was assessed. (D) Binding profile of Suz12 and Pcl3 at the Suz12 locus. Turquoise puncta are background and not statistically significant. (TIF) [file pgen.1002576.s006.tif]
