# Supplementary material for: Polycomb-Like 3 Promotes Polycomb Repressive Complex 2 Binding to CpG Islands and Embryonic Stem Cell Self-Renewal
Source: PLoS Genet. 2012 Mar 15;8(3):e1002576. doi: 10.1371/journal.pgen.1002576 (PMC3305387; doi:10.1371/journal.pgen.1002576)
Supplement: Text S1 — Supplemental methods. Detailed description of the microarray and ChIP-sequencing methods as well as a list of the antibodies and primers used. (DOC) [file pgen.1002576.s009.doc]

**Text S1:**

**Antibodies:** Goat anti-Suz12 (1:500, Santa Cruz sc-46264), rabbit anti-FlagM2 (1:600 for IF and 1:1000 for Western blot, Cell Signaling 2368), mouse anti-α-tubulin (1:5000, Sigma T9026), goat anti-V5 (IF 1:400, Abcam ab9137), rabbit anti-V5 (Western blot 1:5000, Sigma V8137), mouse anti-Ezh2 (1:400, Hamer et al. 2002 Ezh2 M18), mouse anti-Eed (1:1000, gift from Barbara Panning), rabbit anti-β-actin (1:5000, Abcam ab8227), rabbit anti-H3K27me3 (1:3000, Millipore 07-449), mouse anti-H3 (1:5000, Abcam ab10799), mouse anti-H2A119Ub (1:1000, Upstate 05-678 clone E6C5), rabbit anti-H3K9me3 (1:5000, Abcam ab8898), rabbit anti-H3K27ac (1:5000, gift from Barbara Panning), anti-Pcl3 (1:1000, Proteintech), mouse anti-NeuN (1:800, Millipore MAB377), rabbit anti-Tuj1 (1:2000, Covance PRB-435P), mouse anti-Actin (1:100, Sigma A4700), rabbit anti-K14 (1:1000, Covance PRB-155P), mouse anti-E cadherin (1:50, BD Biosciences 610181), rabbit anti-Pcl2 (1:1000, Proteintech), mouse anti-Oct3/4 (C-10) (1:200, Santa Cruz sc-5279), rabbit anti-Nanog (1:200, abcam ab80892), rabbit anti-H3K4me3 (1:1000, Millipore clone MC315 04-745).

**Microarray:** For microarray #2, ESCs were pre-plated twice for one hour on untreated TC plates, counted, and plated at equal densities on gelatinized plates. Microarray #1 was performed similarly but without pre-plating. The next day cells were collected and RNA extracted from two scramble and two *Pcl3* shRNA clones. Total RNA quality was assessed using a Pico Chip on an Agilent 2100 Bioanalyzer. RNA was amplified and labeled with Cy3-CTP using low RNA input fluorescent linear amplification kits following manufacturer’s protocol (Agilent). Concentration of labeled cRNA was assessed using a Nanodrop ND-100, and equal amounts of Cy3 labeled target were hybridized to whole human genome 4x44K Ink-jet arrays (Agilent). Hybridizations were performed for 14 hrs, according to the manufacturer’s protocol (Agilent). Arrays were scanned using an Agilent microarray scanner, and raw signal intensities were extracted with Feature Extraction v10.1 software (Agilent). This dataset was normalized using the *quantile* normalization method that is proposed by Bolstad et al [1]. No background subtraction was performed, and the median feature pixel intensity was used as the raw signal before normalization.

**ChIP-sequencing:** Chromatin immunoprecipitation reactions were performed by washing 50μL of the appropriate Dynal magnetic beads (Dynabeads M-280 Sheep anti-Rabbit IgG, Invitrogen Cat # 112.04D, or Dynabeads M-280 Sheep anti-Mouse IgG, Invitrogen Cat # 112.02D) in 1mL PBS with 5mg/mL BSA three times. Washes were aspirated while tubes were on Dynal MPC-S magnetic strips to retain magnetic beads. Beads were resuspended in 1mL BSA/PBS with 5μg of antibody. The antibodies used were anti-H3K27me3 (Upstate, 07-449), anti-Flag M2 (Sigma, 088k6018), and anti-Suz12 (Upstate, 07-379). After rotating overnight at 4°C, the beads were washed three more times with BSA/PBS and resuspended in 1mL chromatin, 100μL BSA/PBS, 1% Triton-X, 0.1% Sodium Deoxycholate (DOC), 0.1% TE, and 1X EDTA-free Complete (tablets). Samples rotated overnight at 4°C. Beads were then washed 8 times with RIPA buffer (50mM Hepes, pH 8.0, 1mM EDTA, 1% NP-40, 0.7% DOC, 0.5M LiCl, 1X EDTA-free Complete) and washed once with 1X TE. Beads resuspended in 170μL of Elution Buffer (10mM Tris, pH 8.0, 1mM EDTA, 1% SDS) and shaken for 20 minutes at 65°C. Samples were centrifuged at high speed and the supernatant was removed to a new tube and reverse cross-linked overnight at 65°C. 50μL of untreated chromatin was also reverse cross-linked with 120μL Elution Buffer overnight at 65°C. 140μL TE, 7μL Proteinase K, and 3μL Glycogen (20μg/μL) were added to each sample and incubated for 2 hours at 37°C. Samples were extracted twice with 300μL Phenol in a single Phase-Lock tube (5Prime, Cat # 2900308, Lot #42793024), and once with chloroform/isoamyl alcohol. After the aqueous layer was removed to a new tube, NaCl was added to a final concentration of 200mM, followed by 2 volumes of 100% ETOH. Samples were vortexed and incubated at -80°C for 30 minutes, centrifuged for 15 minutes at 15,000 RPM at 4°C, and washed with 1mL 70% ETOH. After centrifuging for 5 minutes at 15,000 RPM at 4°C, pellets were airdried and resuspended in 30μL TE with 10μg RNase A. After incubating 1-2 hours at 37°C, samples were purified using Qiagen MinElute kit and eluted to 34μL with Qiagen EB buffer (50μL for inputs).

**Library Preparation:** 34μL IP samples were treated with End Repair kit (Epicentre, Cat # ER81050, Lot # E85-91206), with 5μL of 10X End Repair buffer, 5μL of 2.5mM dNTPs, 5μL of 10mM ATP, and 1μL of END-IT enzyme mix added to each IP. Samples incubated at room temperature for 45 minutes. Samples were purified using Qiagen MinElute kit and eluted to 32μL. An additional ‘A’ base was added to the 3’ end of DNA fragments by adding 5μL 10X NEBuffer 2 (New England Biolabs, Cat # B7002S, Lot # 0010901), 10μL of 1mM dATP, and 3μL of 5U/μL 3’-5’ exo-Klenow Fragment (New England Biolabs, Cat #M0212L, Lot # 0180912). Samples were incubated at 37°C for 30 minutes and purified with Qiagen MinElute kit, eluting to 20μL. Samples were then evaporated using a speedvac to 4μL, and sequencing adapters were ligated to the ends of the DNA fragments by adding 5μL of 2X Quick Ligase Reaction Buffer (New England Biolabs, Cat # B2200S, Lot # 0010910), 0.5μL of Adapter Oligo mix, 1:10 in H_2_O, and 0.5μL of 1U/μL DNA Ligase (New England Biolabs, Cat # M2200L, Lot # 0980910). Samples were incubated at room temperature for 15 minutes and purified with Qiagen MinElute kit, eluting to 30μL. Samples were evaporated in a speedvac for 2 minutes and loaded with 6X Bromophenol blue/xylene cyanol loading dye on a 15 cm 8% polyacrylamide gel in TBE. 100bp DNA ladder loaded in far right and left lanes. Gel run at 200V for 2 hours and stained with 1X SYBR Gold (Invitrogen Cat # S11494) in 120μL TBE, shaking for 15 minutes. DNA between 200bps and 400bps were excised from gel with a clean scalpel, minced by centrifuging through a sieve, and shook vigorously overnight at 4°C in 500μL of Qiagen EB buffer. The next day, samples were mixed vigorously at 50°C for 15 minutes and centrifuged at 15,000 RPM for 2 minutes. The supernatant was transferred to a 0.2 μm Nanoseq column (Pall, Cat # ODM02C35, Lot # 09551479) and centrifuged at 15,000 RPM for 2 minutes and the flow-through was collected. Repeat if necessary. 1/10 volume of 3M NaOAC (pH 5.2), 4μL of glycogen (20μg/μL), and 2 volumes of 100% ETOH were added to each sample, vortexed, and incubated at -80°C for 30 minutes. Samples centrifuged at 15,000 RPM for 15 minutes at 4°C, washed with 1mL 70% ETOH, and centrifuged again for 5 minutes. Pellets were airdried and resuspended in 20μL of Qiagen EB buffer. 10μL 5X Phusion HF Buffer (Finnzymes, Cat # F-518, Lot # 82), 1μL 10mM dNTPs, 13.5μL PCR grade H_2_O, 0.5μL HotStart Phusion Taq (Finnzymes, Cat # F540L, Lot #40), and 2.5μL each of forward and reverse 10mM Solexa PCR primers were added to each sample, and run with the following PCR program:

Step 1: 98°C for 30 seconds

Step 2: 98°C for 10 seconds

Step 3: 65°C for 30 seconds

Step 4: 72°C for 30 seconds

Step 5: Go to step 2, 17 cycles

Step 6: 72°C for 5 minutes

Step 7: 4°C, forever

Samples purified with Qiagen MinElute kit, eluting to 40μL. Samples were evaporated in speedvac for 2 minutes and loaded on an 8% polyacrylamide gel exactly as before. DNA was excised from same region of gel as before and treated as before. The following day, the samples were treated exactly the same as after the first gel and overnight shaking, except the purified DNA was ultimately resuspended in 10μL of Qiagen EB buffer. Sample DNA concentration was determined using an Invitrogen Qubit Flourimeter and the samples were diluted to 10nM for sequencing.

**Normalization of Paired ChIP-seq Data:** NGS experiments may produce vastly different numbers of total reads for paired data, e.g. Suz12 ChIP after *Pcl3* shRNA vs. scramble, or Suz12 ChIP vs. Input. Suppose there are *M* reads for IP and *N* reads for Input. Then, many researchers currently scale the Input counts per genomic window by *M*/*N*, in order to equalize the total number of reads between the two samples. This method, however, does not take into account that the IP data should accumulate a significantly greater number of reads in regions targeted by the antibody relative to the background; as a result, this scaling approach will artificially inflate the background noise captured by Input. A better alternative approach is to find a scaling factor that equalizes just the background between IP and Input channels without considering the IP peak regions.

In order to separate the antibody-targeted loci from background noise, we will apply the theory of order statistics. Our motivation comes from the following observation for normally distributed random variables: let *Y_1_*, *Y_2_*, ... , *Y_N_* be independent identically distributed normal random variables with mean μ and variance σ^2^, and let *Y*_(1)_, *Y*_(2)_, ... , *Y*(*_N_*) be their order statistics, i.e. the rearrangements of *Y_i_* such that *Y*_(1)_ ≤ *Y*_(2)_ ≤ ... ≤ *Y*(*_N_*). If we partition the reference genome into *N* equal-sized bins, then for sufficiently large μ, we can think of *Y_i_* as counting reads in the *i*-th bin. Define the partial mean $S_{n/N}$ as

$S_{n/N}=\sum_{k=1}^{n} Y_{(k)}/n$.

Because the order statistics are ranked in an increasing order, it can be seen that the partial mean is an increasing function of *n*. In fact, in the limit of large sample size *N*, the partial mean is almost a linear function of *n* with a positive slope. More precisely, for large *N*,

$S_{\alpha}\to\mu-\frac{\sigma}{\alpha}f(Y_{\alpha})$,

where *f* is the probability density of *Y_i_* and α=*n/N* [2]. Expanding this asymptotic form around α=1/2, one can show that the partial sum satisfies

$S_{\alpha}\sim^{N\to\infty} \mu-\frac{\sigma}{\alpha}\frac{(1-\pi(\alpha-1/2)^{2})}{\sqrt{2\pi}}$,

which is **almost linear in α** and can be fitted with linear regression with R^2^ > 0.99.

Similarly, consider a set of bivariate normal random variables *Z_i_* =(*Y_i_*,*X_i_*,), where *Y_i_* are defined as above and *X_1_*,*X_2_*,...,*X_N_* are independent identically distributed normal random variables that are uncorrelated with *Y_i_*. We can think of *Y_i_* as binned immunoprecipitated DNA counts and *X_i_* as binned Input DNA counts. Then, we define the *i*-th order statistic *Z*(*_i_*) to be the pair (*Y_k_*,*X_k_*), such that *Y_k_* corresponds to the *i*-th order statistic *Y*(*_i_*); i.e., the order statistics are obtained by sorting *Z_i_* with respect to the first entry. Because we have assumed that *X* and *Y* are uncorrelated, $\sum_{k=1}^{n} X_{(k)}/n$ is an unbiased estimate of the expectation $E[X]$, and for sufficiently large *n*, the ratio $R_{n/N}=\sum_{k=1}^{n} Y_{(k)}/\sum_{k=1}^{n} X_{(k)}$ of partial sums thus approaches $\sum_{k=1}^{n} Y_{(k)}/nE[X]$, which is proportional to the partial mean $S_{n/N}=\sum_{k=1}^{n} Y_{(k)}/n$. Consequently, for large *N*, the above analysis shows that $R_{n/N}$ can be **approximated by a linear function** of α=*n/N*.

In a more realistic situation, the distribution of the IP channel data *Y_i_* can be modeled as a mixture of two Poisson distributions, e.g. one component representing the basal level of background noise and the second component representing the enrichment of actual immunoprecipitated DNA. For sufficiently large mean, Poisson distributions approach normal distributions, and the above analysis still holds; but, the ratio $R_{\alpha}$ in this case begins to diverge from linearity at α roughly equal to the mixing probability. By computing this critical value of α, we can thus approximate the proportion of background noise in the IP channel data *Y_1_*, *Y_2_*, ... , *Y_N_*. The corresponding ratio$R_{\alpha}$ also provides the optimal scaling factor for normalizing the Input channel *X* in order to equalize the read counts in background regions that are not directly targeted by the antibody. This method can be applied to general paired ChIP-seq data, e.g. Suz12 ChIP-seq with *Pcl3* shRNA vs. scramble. Supplemental Figure 4B provides an example of this method, where the mouse genome was partitioned into 2kb bins.

**Detection of Peaks and Differentially Enriched ChIP-seq Regions:** We have assessed the statistical significance of the differences between paired normalized ChIP-seq data as follows: let n_1_(x) and n_2_(x) be the normalized tag counts in a window centered at genomic location x in Sample 1 and Sample 2, respectively. Then, assuming the null hypothesis that n_1_(x) and n_2_(x) are independent and locally Poisson with common mean λ, the difference *Y*=n_1_(x)-n_2_(x) in tag counts follows the Skellam distribution

$$f(Y=n;\lambda)=e^{-2\lambda}I_{|n|}(2\lambda)$$

where the maximum likelihood estimate (MLE) of the mean is λ=(n_1_(x)+n_2_(x))/2. Because we have only two samples, the MLE may underestimate the true mean and increase the false positive rate. In our analysis, we have estimated the 95% confidence interval and used the more conservative value λ=0.5 qpois(0.975, n_1_(x)+n_2_(x)), where qpois is the quantile function in R for the Poisson random variable n_1_(x)+n_2_(x). For n > 0, the right-tail p-value is


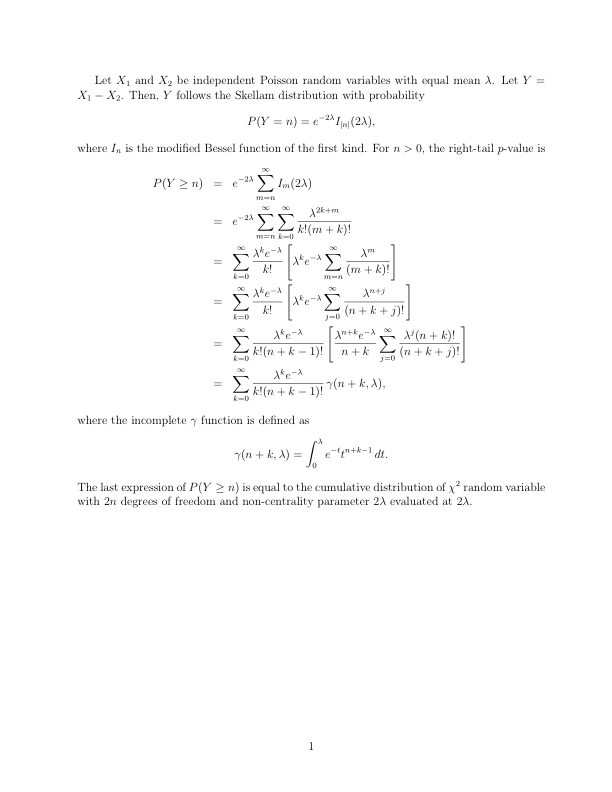


where the incomplete γ function is defined as


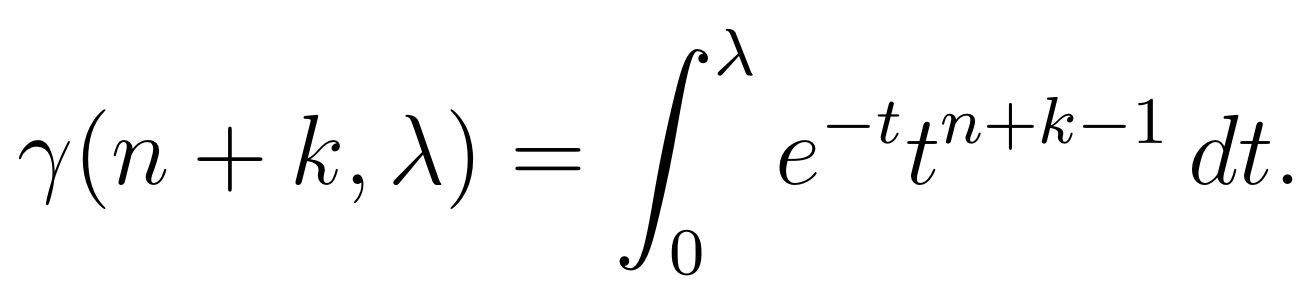


The final expression of the p-value is equal to the cumulative distribution of χ^2^ random variable with 2n degrees of freedom and non-centrality parameter 2λ evaluated at 2λ.

This novel method of detecting differential enrichment is more rubust than other approaches using Poisson *p*-values, which do not take into account the over-dispersion of count data and are thus more prone to false positives.

Primers:

| qRT-PCR primers   |  | | | --- | --- | | *β-actin* | | | CACAGCTTCTTTGCAGCTCCTT | CGTCATCCATGGCGAACTG | | *Suz12* | GTGCACTCTGAACTGCCGTA | |  | CCGGTCCATTTCGACTAAAA | | *Pcl3 set 1* | TGTGTGTACAGCCACCACCT | |  | TGTCCCCAAAGTGATTGTCA | | *Pcl3 set 2* | AGAGGAAGCTGACAGCCAAA | |  | CGTCTCCACTGATGCTTTCA | | *Pcl3-TAP* | AGCACAGACCACCACCTAGC | |  | AATCACCGTCATGGTCTTTG | | *Eed* | CTGGCAAAATGGAGGATGAT | |  | TGGGTCAGTGTTGTGCATTT | | *Ezh2* | AGACGTCCAGCTCCTCTGAA | |  | ATCCTCAGTGGGAACAGGTG | | *Oct4* | CCAATCAGCTTGGGCTAGAG | |  | CTGGGAAAGGTGTCCCTGTA | | *Nanog* | GCTCAGCACCAGTGGAGTATCC | |  | TCCAGATGCGTTCACCAGATAG | | *Igf2* | GTCCCCACATTTGCAGTTCT | |  | CTGGATGACATGGACAGTGG | | *Hand1* | GTTCCCATTCGTTGCTGAAT | |  | CTGCGAGTGGTCACACTGAT | | *Krt7* | ACGGCTGCTGAGAATGAGTT | |  | CGTGAAGGGTCTTGAGGAAG | | *Acta2* | CTGACAGAGGCACCACTGAA | |  | CATCTCCAGAGTCCAGCACA | | *Plac1* | CCTCCATCATGGGACAGAGT | |  | GCCCTTACATCTGGGCACTA | | *Dkk1* | CAGCTCAATCCCAAGGATGT | |  | CAGGGGAGTTCCATCAAGAA | | *Fgf1* | TAAACCCAGGGCTTTCAATG | |  | TTGCAGTCAGCTTGGAATTG | | *Neurod1* | CAAAGCCACGGATCAATCTT | |  | CCCGGGAATAGTGAAACTGA | | *Epha4* | AGGTGGGAGGAGGTCAACTT | |  | GTGAAACGGCTTGATTTGGT | | *Pcl2* | CATTTGCGGAGAAGAAGAGG | |  | TGATTGAGCTGCAACTCCTG | | *Pcl1* | GAGATCCTCCCCTTCACCTC | |  | AAATGAAACGGTCCTTGTGG | | *Meis2* | CATGGTCGTTGCACCATAAG | |  | GTGAGGAACACGTGCTGAGA | | *Meis1* | ACAGGAGACCCGACAATGAG | |  | TTGACTTCGGATGGTTCTCC | | *Cxcl12* | TTTGGCCTCCTGTAGAATGG | |  | TCAGAGCCCATAGAGCCACT | | *Slc23a1* | TGCTCTGCTACGTGTTGACC | |  | GGATCCAGGGAGAGATAGCC | | *Miip* | GGTCACACCACCTCTCTGGT | |  | GGGACAATGGAGGAGAGACA | | *Cbx4* | GCCCTTGAGTGAGTTCAAGC | |  | TCCCACTACACCGTCACGTA | | *Bmp1* | CACTCCACAGCAGGAAGTGA | |  | CTCAGTGAAAGCTCCGGTTC | | *Hoxd10* | GAAGAGGTGCCCTTACACCA | |  | TCGATTCTCTCGGCTCATCT | | *Mia1* | GGGCCAAGTGGTGTATGTCT | |  | GGACAATGCTACTGGGGAAA | | *Manba* | AGTGGACACTGGGGACAAAG | |  | GTCTTCTGGCCGTGCTTTAG | | *Mras* | AGACAGGGCTACAGCTTCCA | |  | GTCTGGTGCGTTGTATGTGG | | *Efhc2* | ACTTTACGCCCATTTCATGC | |  | GCTTCAGGCCTACACAGGAG | | *Zfp42* | CTTCACGGAGAGCTCGAAAC | |  | CTTTGCGTGGGTTAGGATGT | | *Tgfβ1* | TGCTTCAGCTCCACAGAGAA | |  | TGGTTGTAGAGGGCAAGGAC | |  |  | | ChIP primers |  | | *mir196b* | AAGGTGGATCCCAACAACAG | |  | CCTCCTAGGCGTACCTTTCC | | *mir34a* | GGCAATGGCTCAGAGAAGAC | |  | AGCGAGAGCTCAGAGTTTGC | | *mir10a* | CCCTTCCAACTCGTCCATAA | |  | GCCACAGGTTGCTAGTGTGA | | *mir132/212* | GCAGACGCAGACACTATGGA | |  | CTAGGCTGAGTCACCGCTCT | | *mir196a* | GCTGCAGAGGAGATCAAAGG | |  | TCTGCCAAGCAAGAGAAACA | | *Hoxb7* | ACCGAGTTCCTTCAACATGC | |  | CAGGGGTAGATCCGGAAGTT | | *Hoxb13* | CTCGTTAAACCTGCCAAAGC | |  | CATAAAATCCTCCCGCAGAA | | *Pmp22* | CCTAGGACTGCCTCTGCATC | |  | CCAGACACGTCCATTTTCCT | | *Hoxa3* | TCACAGGCTGGTGAGAACAC | |  | AGGGTCATTTGGCAAGACTG | | *Hand1* | GGTGTGAGTGGTGATGATGG | |  | TTTGGACGTCTGAACCCTTC | | *Dusp4* | GCCTCTACTCGGCTGTCATC | |  | CCGCCAAGTCCCTTACACTA | | *Acta1* | TTGTCGATTGTCGTCCTGAG | |  | CAGGCACGTGACACTCTTGT | | *Tle3* | TCCGCAAGGCAGACATCCGGT | |  | CACGCGCGCGCACCATAAAG | | *Tns1* | AGCGGACCCACCACCCTCTG | |  | GGAGTCGGCTGACACGGTGC | | *Tmem151a* | TCCGTGTCCTCCCCTCTGCG | |  | GCCAGCTCTGGGGCCTACCT | | *Eaf1* | CCTCTGGAACTTCGATCTGC | |  | CAAGAGCCCTGATGGAAGAA | | *Ash21* | CAGAGAGCCCGAGGTAGTTG | |  | GCACTCAGTCCAGGGGTTTA | | *Dcaf8* | CCCGATCCCTTTCCTAAAAC | |  | CTAGGAGACGCCAACAGAGC | | *Ift140* | TAGGAGTCCGTCACCGAAAC | |  | AAGCGTGGATGAAAAAGGTG | | *Glrx5* | CGATTTGTCCAATCAACACG | |  | CACTCCAGGGCTTACAGAGC | | *Ebf2* | CCACCCCTGAACTTGTCACT | |  | CTGACGCCTCTTTTCTCACC | | *Mafb2* | AGAAGCGGTCCTCCACACTA | |  | AGCAGGTGTGACTCACGATG | | *Pcdh7* | TTCTTTGGAAATCCGGACAG | |  | GGGAACTCGAGCTGAACTTG | | *Otx2* | TTTCAAAGCGAGAGGGAAGA | |  | CCACGCACATACTGTGGTTC | | *Actin control* | GGAGGTGCAATGGCTGTCTTGTCC | |  | CTGCCTTGGGTCACCTTACACCTCAC | |  |  | | |  |  |
| --- | --- | --- | --- | --- | --- | --- | --- | --- | --- | --- | --- | --- | --- | --- | --- | --- | --- | --- | --- | --- | --- | --- | --- | --- | --- | --- | --- | --- | --- | --- | --- | --- | --- | --- | --- | --- | --- | --- | --- | --- | --- | --- | --- | --- | --- | --- | --- | --- | --- | --- | --- | --- | --- | --- | --- | --- | --- | --- | --- | --- | --- | --- | --- | --- | --- | --- | --- | --- | --- | --- | --- | --- | --- | --- | --- | --- | --- | --- | --- | --- | --- | --- | --- | --- | --- | --- | --- | --- | --- | --- | --- | --- | --- | --- | --- | --- | --- | --- | --- | --- | --- | --- | --- | --- | --- | --- | --- | --- | --- | --- | --- | --- | --- | --- | --- | --- | --- | --- | --- | --- | --- | --- | --- | --- | --- | --- | --- | --- | --- | --- | --- | --- | --- | --- | --- | --- | --- | --- | --- | --- | --- | --- | --- | --- | --- | --- | --- | --- | --- | --- | --- | --- | --- | --- | --- | --- | --- | --- | --- | --- | --- | --- | --- | --- | --- | --- | --- | --- | --- | --- | --- | --- | --- | --- | --- | --- | --- | --- | --- | --- | --- | --- | --- | --- | --- | --- | --- | --- | --- | --- | --- | --- | --- | --- | --- | --- | --- | --- | --- | --- | --- | --- | --- | --- | --- | --- | --- | --- | --- | --- | --- | --- | --- | --- | --- | --- | --- | --- | --- | --- | --- | --- | --- | --- | --- | --- | --- | --- | --- | --- | --- | --- | --- | --- | --- | --- | --- | --- | --- | --- | --- | --- | --- | --- | --- | --- | --- |
|  |  | | |

1. Bolstad BM, Irizarry RA, Astrand M, Speed TP (2003) A comparison of normalization methods for high density oligonucleotide array data based on variance and bias. Bioinformatics 19: 185-193.

2. Burrows PM (1972) Expected selection differentials for directional selection. Biometrics 28: 1091-1100.
